# Millimeter Scale Track Irregularity Surveying Based on ZUPT-Aided INS with Sub-Decimeter Scale Landmarks

**DOI:** 10.3390/s17092083

**Published:** 2017-09-12

**Authors:** Qingan Jiang, Wenqi Wu, Yun Li, Mingming Jiang

**Affiliations:** Department of Automatic Control, College of Mechatronics and Automation, National University of Defense Technology, Changsha 410073, China; jqa1987@nudt.edu.cn (Q.J.); liyun2009@nudt.edu.cn (Y.L.); uninavi@163.com (M.J.)

**Keywords:** railway track irregularity, INS, ZUPT, Kalman filtering, RTS smoothing, covariance analysis

## Abstract

Railway track irregularity surveying is important for the construction and the maintenance of railway lines. With the development of inertial devices, systems based on Inertial Navigation System (INS) have become feasible and popular approaches in track surveying applications. In order to overcome the requirement of high precision control points, this paper proposes a railway track irregularity measurement approach using the INS combined with the Zero Velocity Updates (ZUPT) technique and sub-decimeter scale landmarks. The equations for calculating track irregularity parameters from absolute position errors are deduced. Based on covariance analysis, the analytical relationships among the track irregularity measurements with the drifts of inertial sensors, the initial attitude errors and the observations of velocity and position are established. Simulations and experimental results show that the relative accuracy for 30 m chord of the proposed approach for track irregularity surveying can reach approximately 1 mm (1*σ*) with gyro bias instability of 0.01°/h, random walk noise of 0.005${}^{\circ}/\sqrt{h}$, and accelerometer bias instability of 50 μg, random noise of 10 $\mu g/\sqrt{\mathrm{Hz}}$, while velocity observations are provided by the ZUPT technique at about every 60 m intervals. This accuracy can meet the most stringent requirements of millimeter scale medium wavelength track irregularity surveying for railway lines. Furthermore, this approach reduces the requirement of high precision landmarks which can lighten the maintenance burden of control points and improve the work efficiency of railway track irregularity measurements.

## 1. Introduction

Railway track irregularity is one of the most important factors affecting the safe operation of the train. The irregularity can be assessed by track geometry parameters, the measurement of which plays a significant role in monitoring the track deformation and guiding the maintenance of railway lines [1]. Trains with higher speed require higher track smoothness. With the development of high-speed railways, the demands for track irregularity measurement techniques with high-accuracy and high-efficiency are increasing rapidly [2].

Traditionally, there are mainly two categories of track irregularity measurement methods namely dynamic measurement and static measurement. Methods based on Track Recording Coaches (TRCs) are a kind of dynamic one under wheel loading [1,2]. TRC can measure long wavelength track irregularities with high work efficiency, but their availability is restricted and the measuring accuracy does not fulfil the requirements for track renewals [2]. Another method based on manual measuring devices is a kind of static one. These kinds of devices used for spot assessment are surpassed by railway track surveying trolleys in terms of data amounts and time efficiency. The lightweight and flexible track surveying trolleys can handle different measurement tasks such as measurements during the construction stage and measurements of shorter stretches of track [1,2].

The most well-known track surveying trolleys, widely deployed in China, are the GPR1000 series products provided by Amberg Technologies (Regensdorf, Switzerland) [3]. These kinds of trolleys are all-in-one solutions for surveying railway tracks, enabling the assessment of parameters such as the cant, track gauge, chainage, alignment and level with high accuracy, and can provide sub-millimeter absolute accuracy measured by total station in stop-and-go mode. The Swiss Trolley is another representative track surveying trolley which is developed by the Institute of Geodesy and Photogrammetry at ETH Zurich [1,2]. Glaus [2] has presented this kind of trolley thoroughly. The basic devices the Swiss Trolley is equipped with are an inclinator, track gauge measuring system and odometer can measure gradient, cant, gauge, and chainage. Absolute positioning is done by Global Positioning System (GPS) and total station. According to Glaus [2], GPS measurements fulfill most accuracy requirements contrary to the general opinion that submillimeter absolute accuracy has to be obtained in railway surveying.

With the development of inertial sensors, INS is no longer expensive and bulky. Track surveying systems based on INS have been widely applied in railway track surveying. A limitation of a stand-alone INS is its unfavourable error propagation. Error drifts of INS should be depressed by other sensors providing relative or absolute measurement updates such as ZUPT, Control Points Coordinate Updates (CUPT) and so on. Luck [4] discussed the design of track measurement systems based on INS/GPS integration for the dynamic inspection locomotive. Niu and Chen [3] presented an INS/GPS integrated system to measure railway track irregularities with relative accuracy of 1 mm. Non-holonomic constraint and ZUPT are implemented in their integration algorithm to improve the surveying accuracy. In order to implement the measuring task with GPS outages, Li [5] presented a track irregularity measurement trolley equipped with a laser-aided INS/odometer integration system for subway applications whose observations of control points for position updates are measured by the laser scanner. The standard deviations of alignment and vertical irregularities can reach approximately 1 mm. Jiang [6] utilized the Inertial Measurement Unit (IMU)/odometer/landmark integration technique for railway track surveying and obtained absolute accuracy of 1 mm.

The key issue about INS-based track irregularity surveying systems is suppressing the position errors produced by drifts of inertial sensors using global position information [5]. In previous works, a great job about the integrations of INS with position measurement sensors and the data fusion of INS measurement with position information has been done for track surveying, but there are few studies about the accuracy requirement of inertial sensors and observation updates that can meet the demands for railway irregularity measurement theoretically or experimentally. In addition, the traditional measurement approaches based on global position information have their own shortcomings. The GPS signal may be disturbed by obstructions which affect the GPS solutions negatively. The distribution and precision of control points are crucial to ensure measurement accuracy for approaches based on landmarks. The construction of control points is costly and their maintenance at millimeter scale is difficult. Even if high precision landmarks can be maintained well, high precise position observations cannot be provided frequently in order to insure the work efficiency of the measurement task, especially for environments without GPS signals, for instance tunnels. Therefore, it is significant to research approaches using less number of landmarks with low precision for railway track irregularity measurement.

This paper focuses on the issue of the railway track irregularity measurement using a ZUPT-aided INS to achieve high accuracy measurement of relative track geometry parameters to reduce the requirement of high precision landmarks. A typical Kalman filter with 12 dimensional error states is designed in the paper and the Rauch-Tung-Striebel (RTS) smoother is employed to improve the position accuracy. Aiming at alignment and level irregularities of the track, we established the relationship between track irregularities and the absolute position deviations. Based on covariance analysis, the surveying accuracy of alignment and level irregularities is presented, the analytical relationship about irregularities with the precisions of inertial sensors, initial attitudes and observation updates are established. Simulation and experimental results are also presented.

The rest of the paper is organized as follows: Section 2 describes the railway track irregularity and assessment. Section 3 describes the overview of the measurement system and the algorithm. Section 4 describes the design of Kalman filter and smoother. Section 5 presents the calculation method of alignment and level irregularities from absolute position as well as the covariance analysis of them. Section 6 reports the simulation and experimental results of track irregularity. Section 7 concludes this paper.

## 2. Railway Track Irregularity and Assessment

**Figure 1:**
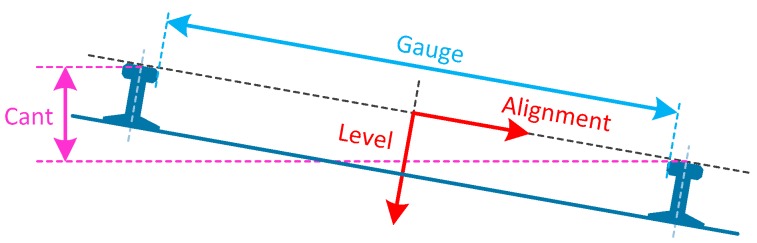
Track coordinate system and geometry parameters.

Railway tracks can be regarded as a 3-dimensional curve [3,5]. Track irregularity refers to the deviation of the track from its design geometry, which is usually determined by five geometry parameters, namely alignment (horizontal alignment), level (vertical alignment), cant (super-elevation or cross-level), twist and gauge [7,8,9]. As illustrated in Figure 1, the axes of the rail coordinate system are defined as follows: the *x*-axis is in the travelling direction, *y*-axis parallel to the running surface, and *z*-axis perpendicular to the running surface and pointing downwards. Alignment is the track’s displacement in the horizontal plane, which can be seen as the deviation of actual track from the design one in horizontal plane. Level is the displacement in the vertical plane [1]. Gauge is the distance between the inner sides of the two railheads. Cant is the difference between the elevations of the running surface of two rails, representing the tilting of the track in curves in order to compensate the centripetal force. Twist is defined as the difference in cant over a given length.

In this paper, the gauges are estimated from track gauge measurement system. Cant and twist can be calculated in simple models, and will not be discussed in detail. The alignment and level irregularities will be evaluated as examples to demonstrate the measuring accuracy.

**Figure 2:**
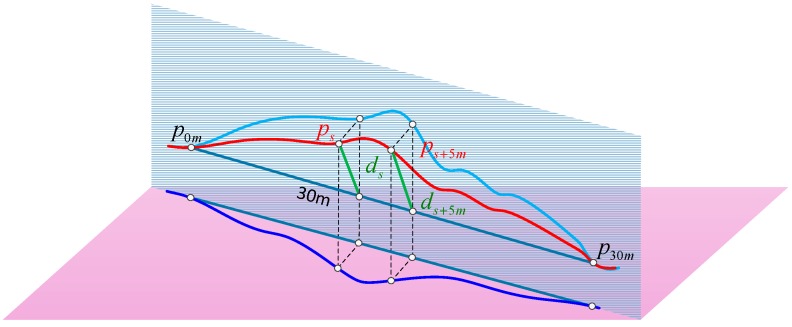
Vector distances of two points with 5 m interval on a 3D curve for 30 m chord.

According to the railway standards [8,9], the alignment and level are measured by the vector distance value with a chord of fixed length (e.g., 30 m) on the rail surface in the horizontal and vertical directions respectively. The magnitude of alignment and level irregularities will be calculated by differential method of 30 m chord. As shown in Figure 2, the red curve represents a segmentation of railway track in 3-dimensional space; the other two curves are the projections in horizontal plane and vertical plane respectively. The 30 m long chord is determined by points $p_{0m}$ and $p_{30m}$ on the curve. Take the point $p_{s}$ on the curve as an example. The distance from $p_{s}$ to the chord is the vector distance of this point represented by $d_{s}$. Vector distance of the next adjacent point $p_{s+5m}$ with 5 m interval is $d_{s+5m}$. The track irregularity of point $p_{s}$ can be calculated by Equation (1) [8]:

(1) $$\Delta_{s}=\left(d_{s}-d_{s+5m}\right)-\left({\tilde{d}}_{s}-{\tilde{d}}_{s+5m}\right)=\left(d_{s}-{\tilde{d}}_{s}\right)-\left(d_{s+5m}-{\tilde{d}}_{s+5m}\right)$$

where ${\tilde{d}}_{s}$ and ${\tilde{d}}_{s+5m}$ represent measurement values of vector distances about points $p_{s}$ and $p_{s+5m}$. $d_{s}$ and $d_{s+5m}$ represent the design values of them. $\Delta_{s}$ represents the track irregularity of point $p_{s}$, projection of which in the horizontal plane is the alignment irregularity and in the vertical plane is the level irregularity.

## 3. Track Irregularity Measurement System

**Figure 3:**
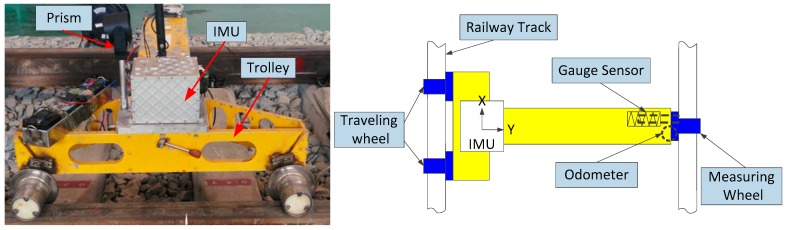
The track irregularity measurement system and its configuration diagram.

The track irregularity measurement system is illustrated in Figure 3. The system is equipped with a T-type trolley, a track gauge sensor, a high precision prism, an odometer, and a navigation grade IMU. The IMU consists of three high accuracy ring laser gyros (RLGs, bias instability: 0.01°/h and angular random walk (ARW): 0.005${}^{\circ}/\sqrt{h}$) and three high stability quartz accelerometers (bias instability: 50 μg and random noise: 10 $\mu g/\sqrt{\mathrm{Hz}}$). The prism mounted on the trolley is used to provide position observations worked with a Leica optical total station (1 mm and 0.5″) based on control points. The odometer of the system can be used as an aid to determine the position of the irregularity measurements along the track in this paper. The gauge sensor is used to measure the gauge of the tracks.

## 4. Kalman Filtering and Smoothing Algorithm Design

### 4.1. Overview of Data Processing

**Figure 4:**
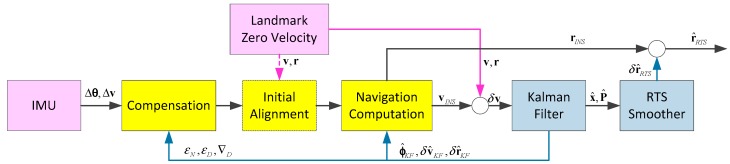
Block diagram of the filtering and smoothing algorithm.

Figure 4 illustrates an overview of the data processing procedure of the Kalman filtering and smoothing algorithm based on ZUPT-aided INS combined with landmarks employed in the paper. The system makes use of the measurements of IMU (angular increments Δθ from gyros and specific force integrations Δv from accelerometers) and the initial position measured by total station for initial alignment to calculate the initial attitudes. After that the trolley is pushed forward manually on the track at walking speed. After it moves across a certain distance interval (60 m in this paper), the trolley stops and a zero-velocity observation and a position observation will be updated. Then Kalman filtering and smoothing algorithm is executed to output the optimized position, velocity and attitude measurements of the interval. Since the wheels of the trolley can keep continuous contact with the railway track, the 3-dimensional track geometry can be determined by position and attitude sequences of the INS uniquely. Then track parameters can be calculated and track irregularities can be detected.

### 4.2. System Equations and Measurement Equations of Kalman Filter

The error equations of the attitude, velocity and position for the railway track surveying application can be expressed as Equation (2) [10]:

(2) $$\begin{array}{ll} \phi^{n} & =-\omega_{in}^{n}\times \phi^{n}+\delta \omega_{in}^{n}-C_{b}^{n}\delta \omega_{ib}^{b}\\ & \approx -\omega_{ie}^{n}\times \phi^{n}-C_{b}^{n}\delta \omega_{ib}^{b}\\ \delta {\dot{v}}^{n} & =f^{n}\times \phi^{n}-\left(2\omega_{ie}^{n}+\omega_{en}^{n}\right)\times \delta v^{n}-\left(2\delta \omega_{ie}^{n}+\delta \omega_{en}^{n}\right)\times v^{n}+C_{b}^{n}\delta f^{b}\\ & \approx f^{n}\times \phi^{n}-2\omega_{ie}^{n}\times \delta v^{n}+C_{b}^{n}\delta f^{b}\\ \delta {\dot{r}}^{n} & =\delta v^{n} \end{array}$$

where i-frame is the inertial frame. n-frame is the local level frame (North-East-Down) used as the navigation frame. *b*-frame is the body frame of the IMU (Forward-Right-Down). $\phi^{n}={\left[\begin{array}{lll} \phi_{N} & \phi_{E} & \phi_{D} \end{array}\right]}^{T}$ represents the vector of attitude errors about the north, east and downward axes of the navigation frame. $C_{b}^{n}$ represents the direction cosine matrix. $\omega_{in}^{n}$ represents the turn rate of the navigation frame with respect to the inertial frame expressed in the *n*-frame. It can be obtained by summing the Earth’s rotation rate with respect to the inertial frame and the turn rate of the navigation frame with respect to the Earth as: $\omega_{in}^{n}=\omega_{ie}^{n}+\omega_{en}^{n}$. $\delta \omega_{ib}^{b}$ represents the drift errors of gyroscopes. $\delta v^{n}$ is the vector of velocity errors. $f^{n}$ represents the specific force in navigation axes. $\delta f^{b}$ is the drift errors of accelerometers. $\delta r^{n}$ represents the position error in navigation axes. The errors of inertial sensors in this paper are normally modeled as piecewise constant values. The position coordinates of the measured track are expressed in segmentation with $n_{0}$-frame, which is so near with the n-frame that $C_{n}^{n_{0}}\approx I$ and $\delta r^{n_{0}}=C_{n}^{n_{0}}\delta r^{n}\approx \delta r^{n}={\left[\begin{array}{lll} \delta r_{N} & \delta r_{E} & \delta r_{D} \end{array}\right]}^{T}$. For medium wavelength (30 m chord) track irregularity surveying, the track segmentation is set to be 60 m long in this paper. Moreover $\delta \omega_{ib}^{b}$ and $\delta f^{b}$ can be expressed as shown by Equations (3) and (4):

(3) $$C_{b}^{n}\delta \omega_{ib}^{b}=\delta \omega_{ib}^{n}={\left[\begin{array}{lll} \varepsilon_{N} & \varepsilon_{E} & \varepsilon_{D} \end{array}\right]}^{T}+C_{b}^{n}{\left[\begin{array}{lll} w_{gx} & w_{gy} & w_{gz} \end{array}\right]}^{T}$$

where $\delta \omega_{ib}^{n}$ is the drifts of gyros expressed in n-frame. $\varepsilon_{N}$, $\varepsilon_{E}$ and $\varepsilon_{D}$ are the equivalent gyro biases of the north, east and downward directions. $w_{gx}$, $w_{gy}$ and $w_{gz}$ are the random noises of gyros:

(4) $$C_{b}^{n}\delta f^{b}=\delta f^{n}={\left[\begin{array}{lll} \nabla_{N} & \nabla_{E} & \nabla_{D} \end{array}\right]}^{T}+C_{b}^{n}{\left[\begin{array}{lll} w_{ax} & w_{ay} & w_{az} \end{array}\right]}^{T}$$

where $\delta f^{n}$ is the drifts of accelerometers expressed in n-frame. $\nabla_{N}$, $\nabla_{E}$ and $\nabla_{D}$ are the equivalent accelerometer biases of the north, east and downward directions. $w_{ax}$, $w_{ay}$ and $w_{az}$ are the random noises of accelerometers.

The railway track surveying application has its own characteristics compared with some other applications based on inertial measurement such as land vehicle navigation, airborne gravity measurement and so on. Since the track of high speed railways is almost level and straight with a very large radius of curvature, trolley maneuvers are rather weak when moving on the track at low speed [3,6]. Some error parameters of the INS are coupled together with others, for example, the orientation error is coupled with the equivalent east gyro bias, and the level errors are coupled with equivalent horizontal accelerometer biases, so the equivalent east gyro bias $\varepsilon_{E}$ and the equivalent horizontal accelerometer biases $\nabla_{N}$ and $\nabla_{E}$ are unobservable. They will not be estimated as error states in the Kalman filter. Since the trolley moves in walking speed (less than 8 km/h [8]) and the length of the measurement interval is short, the terms of $\omega_{en}^{n}$ and $\delta \omega_{in}^{n}$ can be ignored as shown in Equation (2).

Consider the analysis above, a typical Kalman filter with 12 dimensional error states is established in this paper. The system error model and the observation model can be expressed as Equation (5):

(5) $$\begin{array}{l} \dot{x}\left(t\right)=Ax\left(t\right)+Gw\left(t\right)\\ z\left(t\right)=Hx\left(t\right)+\upsilon \left(t\right) \end{array}$$

where the error state vector x(t) can be written as in Equation (6):

(6) $$x\left(t\right)={\left[\begin{array}{llllllllllll} \phi_{N} & \phi_{E} & \phi_{D} & \delta v_{N} & \delta v_{E} & \delta v_{D} & \delta r_{N} & \delta r_{E} & \delta r_{D} & \varepsilon_{N} & \varepsilon_{D} & \nabla_{D} \end{array}\right]}^{T}$$

According to Equation (2), the system error matrix A and the system noise matrix G can be expressed in simplified form by Equations (7) and (8):

(7) $$A=\left[\begin{array}{cccccccccccc} 0 & -\Omega \sin L & 0 & 0 & 0 & 0 & 0 & 0 & 0 & -1 & 0 & 0\\ \Omega \sin L & 0 & \Omega \cos L & 0 & 0 & 0 & 0 & 0 & 0 & 0 & 0 & 0\\ 0 & -\Omega \cos L & 0 & 0 & 0 & 0 & 0 & 0 & 0 & 0 & -1 & 0\\ 0 & -f_{D} & f_{E} & 0 & -2\Omega \sin L & 0 & 0 & 0 & 0 & 0 & 0 & 0\\ f_{D} & 0 & -f_{N} & 2\Omega \sin L & 0 & 2\Omega \cos L & 0 & 0 & 0 & 0 & 0 & 0\\ -f_{E} & f_{N} & 0 & 0 & -2\Omega \cos L & 0 & 0 & 0 & 0 & 0 & 0 & 1\\ 0 & 0 & 0 & 1 & 0 & 0 & 0 & 0 & 0 & 0 & 0 & 0\\ 0 & 0 & 0 & 0 & 1 & 0 & 0 & 0 & 0 & 0 & 0 & 0\\ 0 & 0 & 0 & 0 & 0 & 1 & 0 & 0 & 0 & 0 & 0 & 0\\ 0 & 0 & 0 & 0 & 0 & 0 & 0 & 0 & 0 & 0 & 0 & 0\\ 0 & 0 & 0 & 0 & 0 & 0 & 0 & 0 & 0 & 0 & 0 & 0\\ 0 & 0 & 0 & 0 & 0 & 0 & 0 & 0 & 0 & 0 & 0 & 0 \end{array}\right]$$

(8) $$G=\left[\begin{array}{cc} -C_{b}^{n} & 0_{3\times 3}\\ 0_{3\times 3} & C_{b}^{n} \end{array}\right]$$

$z\left(t\right)={\left[\begin{array}{ll} \delta v^{n} & \delta r^{n} \end{array}\right]}^{T}$ is the filter observation vector and velocity as well as position of the trolley are used as update information in the Kalman filter. The measurement matrix H is defined by Equation (9):

(9) $$H=\left[\begin{array}{cccc} 0_{3\times 3} & I_{3\times 3} & 0_{3\times 3} & 0_{3\times 3}\\ 0_{3\times 3} & 0_{3\times 3} & I_{3\times 3} & 0_{3\times 3} \end{array}\right]$$

w(t) and υ(t) are the system noise and the measurement noise, whose Power Spectral Density (PSD) are Q(t) and R(t) respectively. They can be expressed as Equation (10):

(10) $$w\left(t\right)={\left[\begin{array}{llllll} w_{gx} & w_{gy} & w_{gz} & w_{ax} & w_{ay} & w_{az} \end{array}\right]}^{T},\;w\left(t\right)∼N\left(0,Q\left(t\right)\right)\;Q\left(t\right)=diag\left(\left[\begin{array}{llllll} \sigma_{w_{gx}}^{2} & \sigma_{w_{gy}}^{2} & \sigma_{w_{gz}}^{2} & \sigma_{w_{ax}}^{2} & \sigma_{w_{ay}}^{2} & \sigma_{w_{az}}^{2} \end{array}\right]\right)\;\upsilon \left(t\right)={\left[\begin{array}{llllll} \upsilon_{v_{N}} & \upsilon_{v_{E}} & \upsilon_{v_{D}} & \upsilon_{r_{N}} & \upsilon_{r_{E}} & \upsilon_{r_{D}} \end{array}\right]}^{T},\;\upsilon \left(t\right)∼N\left(0,R\left(t\right)\right)\;R\left(t\right)=diag\left(\left[\begin{array}{llllll} \sigma_{v_{N}}^{2} & \sigma_{v_{E}}^{2} & \sigma_{v_{D}}^{2} & \sigma_{r_{N}}^{2} & \sigma_{r_{E}}^{2} & \sigma_{r_{D}}^{2} \end{array}\right]\right)$$

### 4.3. Smoothing Algorithm

**Figure 5:**
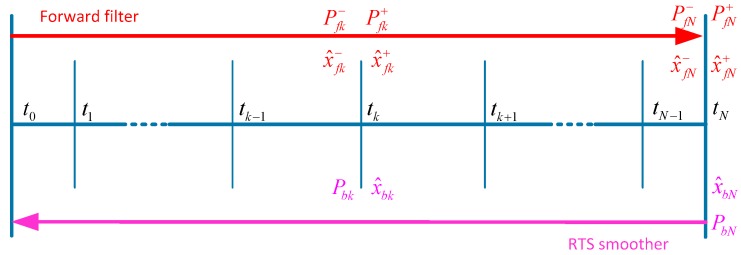
The RTS smoothing algorithm computational process.

The position errors and their covariance between two observation updates will increase with time caused by the residual system errors. It is even more serious for the situations that the observation is few and not much precise. In order to obtain optimal position estimations during the updates outages, a smoothing algorithm must be applied utilizing all the past, current and future measurements [11,12]. This paper employs the well-known RTS smoothing algorithm to estimate the states in the measurement intervals. The RTS smoother consists of a common forward Kalman filter and a backward smoother. The backward sweep begins at the end of the forward Kalman filter. Figure 5 illustrates the computation procedure of the RTS smoother.

The forward Kalman filter is the common one, can be expressed in discrete form as Equation (11) shows:

(11) $$\begin{array}{l} K_{k}=P_{fk}^{-}H_{k}^{T}{\left[H_{k}P_{fk}^{-}H_{k}^{T}+R_{k}\right]}^{-1}\\ {\hat{x}}_{fk}^{+}={\hat{x}}_{fk}^{-}+K_{k}\left[{\tilde{z}}_{k+1}-H_{k}{\hat{x}}_{fk}^{-}\right]\\ P_{fk}^{+}=\left[I-K_{k}H_{k}\right]P_{fk}^{-}\\ {\hat{x}}_{fk+1}^{-}=\Phi_{k}{\hat{x}}_{fk}^{+}\\ P_{fk+1}^{-}=\Phi_{k}P_{fk}^{+}\Phi_{k}^{T}+\Gamma_{k}Q_{k}\Gamma_{k}^{T} \end{array}$$

where ${\hat{x}}_{fk}^{+}$ and $P_{fk}^{+}$ represent the updated estimate of state vector and its corresponding covariance matrix of the forward filter at epoch *k*. ${\hat{x}}_{fk+1}^{-}$ is the optimal predicted estimate and $P_{fk+1}^{-}$ represents its covariance matrix. $H_{k}$ is the measurement matrix. $K_{k}$ is the gain matrix of forward Kalman filter at epoch *k*. $\Phi_{k}$ is the system state transition matrix which can be calculated by matrix A.

The backward smoother can be expressed in the discrete form as shown by Equation (12) [13,14]:

(12) $$\begin{array}{l} {\hat{x}}_{N}={\hat{x}}_{fN}^{+},P_{N}=P_{fN}^{+}\\ {\overset{⌢}{H}}_{k}=P_{fk}^{+}\Phi_{k}^{T}{\left(P_{fk+1}^{-}\right)}^{-1}\\ {\hat{x}}_{k}={\hat{x}}_{fk}^{+}+{\overset{⌢}{H}}_{k}\left[{\hat{x}}_{k+1}-{\hat{x}}_{fk+1}^{-}\right]\\ P_{k}=P_{fk}^{+}-{\overset{⌢}{H}}_{k}\left[P_{fk+1}^{-}-P_{k+1}\right]{\overset{⌢}{H}}_{k}^{T} \end{array}$$

where ${\hat{x}}_{k}$ is the optimal smoothed estimate of state vector at time epoch *k*. $P_{k}$ is the error state covariance matrix of the smoother. ${\overset{⌢}{H}}_{k}$ is the smoothing gain matrix. 

### 4.4. Alignment Irregularity and Level Irregularity Calculated from Absolute Poisition Devition

**Figure 6:**
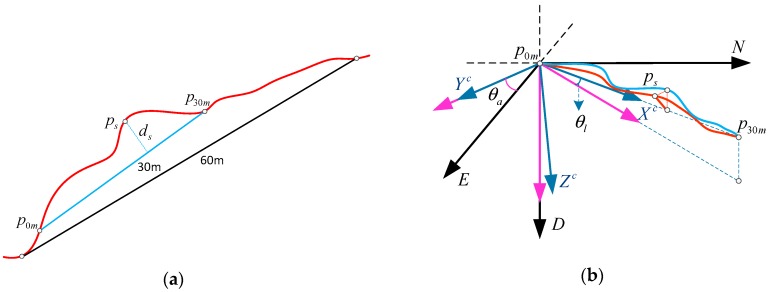
(**a**) Track segmentation and the 30m chord; (**b**) Alignment and level calculated from the navigation coordinate.

In order to assess the track irregularity, the relative geometry parameters of alignment and level should be calculated after obtaining the absolute position of the track. As Figure 6 illustrates, an arbitrarily 30 m chord on the 60 m track segmentation is determined by two points $p_{0m}$ and $p_{30m}$ whose coordinates are respectively marked in $n_{0}$-frame as $r_{p_{0m}}^{n_{0}}={\left[\begin{array}{lll} r_{p_{0m}N} & r_{p_{0m}E} & r_{p_{0m}D} \end{array}\right]}^{T}$ and $r_{p_{30m}}^{n_{0}}={\left[\begin{array}{lll} r_{p_{30m}N} & r_{p_{30m}E} & r_{p_{30m}D} \end{array}\right]}^{T}$. The measurement values of their coordinates are marked as ${\tilde{r}}_{p_{0m}}^{n_{0}}={\left[\begin{array}{lll} {\tilde{r}}_{p_{0m}N} & {\tilde{r}}_{p_{0m}E} & {\tilde{r}}_{p_{0m}D} \end{array}\right]}^{T}$ and ${\tilde{r}}_{p_{30m}}^{n_{0}}={\left[\begin{array}{lll} {\tilde{r}}_{p_{30m}N} & {\tilde{r}}_{p_{30m}E} & {\tilde{r}}_{p_{30m}D} \end{array}\right]}^{T}$ respectively. $p_{s}$ represents an arbitrary point on the trajectory for this chord, coordinates of which are marked as $r_{p_{s}}^{n_{0}}={\left[\begin{array}{lll} r_{p_{s}N} & r_{p_{s}E} & r_{p_{s}D} \end{array}\right]}^{T}$ for the true value and ${\tilde{r}}_{p_{s}}^{n_{0}}={\left[\begin{array}{lll} {\tilde{r}}_{p_{s}N} & {\tilde{r}}_{p_{s}E} & {\tilde{r}}_{p_{s}D} \end{array}\right]}^{T}$ for the measurement value. $d_{s}$ is the vector distance from $p_{s}$ to the chord, projections of which in horizontal plane and vertical plane are alignment and level, respectively.

As shown in Figure 6, the track segmentation is 60 m long for ZUPT and absolute position update. For arbitrary 30 m chord on the track segmentation, we define a new frame as c-frame, whose *x*-axis is identical with the chord and can be obtained by a rotation $\theta_{a}$ about *z*-axis and a rotation $\theta_{l}$ about *y*-axis of $n_{0}$-frame sequentially. We can calculate the vector distance values for every point of a trajectory by transforming the coordinates from $n_{0}$-frame to c-frame. The relationship between $n_{0}$-frame and c-frame can be written as Equation (13):

(13) $$\begin{array}{ll} r_{p_{s}}^{c} & =\left[\begin{array}{c} r_{p_{s}x}^{c}\\ r_{p_{s}y}^{c}\\ r_{p_{s}z}^{c} \end{array}\right]=\left[\begin{array}{ccc} \cos\theta_{l} & 0 & -\sin\theta_{l}\\ 0 & 1 & 0\\ \sin\theta_{l} & 0 & \cos\theta_{l} \end{array}\right]\left[\begin{array}{ccc} \cos\theta_{a} & \sin\theta_{a} & 0\\ -\sin\theta_{a} & \cos\theta_{a} & 0\\ 0 & 0 & 1 \end{array}\right]\left[\begin{array}{c} r_{p_{s}N}^{n_{0}}-r_{p_{0m}N}^{n_{0}}\\ r_{p_{s}E}^{n_{0}}-r_{p_{0m}E}^{n_{0}}\\ r_{p_{s}D}^{n_{0}}-r_{p_{0m}D}^{n_{0}} \end{array}\right]\\ & =\left[\begin{array}{ccc} \cos\theta_{l}\cos\theta_{a} & \cos\theta_{l}\sin\theta_{a} & -\sin\theta_{l}\\ -\sin\theta_{a} & \cos\theta_{a} & 0\\ \sin\theta_{l}\cos\theta_{a} & \sin\theta_{l}\sin\theta_{a} & \cos\theta_{l} \end{array}\right]\left[\begin{array}{c} r_{p_{s}N}^{n_{0}}-r_{p_{0m}N}^{n_{0}}\\ r_{p_{s}E}^{n_{0}}-r_{p_{0m}E}^{n_{0}}\\ r_{p_{s}D}^{n_{0}}-r_{p_{0m}D}^{n_{0}} \end{array}\right] \end{array}$$

According to the definition of alignment and level together with the Figure 6, $r_{p_{s}y}^{c}$ represents the alignment and $r_{p_{s}z}^{c}$ represents the level and they can be calculated by Equation (14):

(14) $$\begin{array}{l} r_{p_{s}y}^{c}=-\left(r_{p_{s}N}^{n_{0}}-r_{p_{0m}N}^{n_{0}}\right)\sin\theta_{a}+\left(r_{p_{s}E}^{n_{0}}-r_{p_{0m}E}^{n_{0}}\right)\cos\theta_{a}\\ r_{p_{s}z}^{c}=\left(r_{p_{s}N}^{n_{0}}-r_{p_{0m}N}^{n_{0}}\right)\sin\theta_{l}\cos\theta_{a}+\left(r_{p_{s}E}^{n_{0}}-r_{p_{0m}E}^{n_{0}}\right)\sin\theta_{l}\sin\theta_{a}+\left(r_{p_{s}D}^{n_{0}}-r_{p_{0m}D}^{n_{0}}\right)\cos\theta_{l} \end{array}$$

In addition, we can express $\theta_{a}$ and $\theta_{l}$ by coordinate values as shown in Equation (15) according to Figure 6:

(15) $$\begin{array}{l} \sin\theta_{a}=\frac{r_{p_{30m}E}^{n_{0}}-r_{p_{0m}E}^{n_{0}}}{\sqrt{{\left(r_{p_{30m}N}^{n_{0}}-r_{p_{0m}N}^{n_{0}}\right)}^{2}+{\left(r_{p_{30m}E}^{n_{0}}-r_{p_{0m}E}^{n_{0}}\right)}^{2}}}，\cos\theta_{a}=\frac{r_{p_{30m}N}^{n_{0}}-r_{p_{0m}N}^{n_{0}}}{\sqrt{{\left(r_{p_{30m}N}^{n_{0}}-r_{p_{0m}N}^{n_{0}}\right)}^{2}+{\left(r_{p_{30m}E}^{n_{0}}-r_{p_{0m}E}^{n_{0}}\right)}^{2}}}\\ \sin\theta_{l}=\frac{r_{p_{0m}D}^{n_{0}}-r_{p_{30m}D}^{n_{0}}}{\sqrt{{\left(r_{p_{30m}N}^{n_{0}}-r_{p_{0m}N}^{n_{0}}\right)}^{2}+{\left(r_{p_{30m}E}^{n_{0}}-r_{p_{0m}E}^{n_{0}}\right)}^{2}+{\left(r_{p_{30m}D}^{n_{0}}-r_{p_{0m}D}^{n_{0}}\right)}^{2}}}\\ \cos\theta_{l}=\frac{\sqrt{{\left(r_{p_{30m}N}^{n_{0}}-r_{p_{0m}N}^{n_{0}}\right)}^{2}+{\left(r_{p_{30m}E}^{n_{0}}-r_{p_{0m}E}^{n_{0}}\right)}^{2}}}{\sqrt{{\left(r_{p_{30m}N}^{n_{0}}-r_{p_{0m}N}^{n_{0}}\right)}^{2}+{\left(r_{p_{30m}E}^{n_{0}}-r_{p_{0m}E}^{n_{0}}\right)}^{2}+{\left(r_{p_{30m}D}^{n_{0}}-r_{p_{0m}D}^{n_{0}}\right)}^{2}}} \end{array}$$

The deviations of $r_{p_{s}y}^{c}$ and $r_{p_{s}z}^{c}$ can be calculated by variational method as expressed by Equations (16) and (17):

(16) $$\delta r_{p_{s}y}^{c}=\frac{\partial r_{p_{s}y}^{c}}{\partial r_{p_{s}N}^{n_{0}}}\delta r_{p_{s}N}^{n_{0}}+\frac{\partial r_{p_{s}y}^{c}}{\partial r_{p_{0m}N}^{n_{0}}}\delta r_{p_{0m}N}^{n_{0}}+\frac{\partial r_{p_{s}y}^{c}}{\partial r_{p_{s}E}^{n_{0}}}\delta r_{p_{s}E}^{n_{0}}+\frac{\partial r_{p_{s}y}^{c}}{\partial r_{p_{0m}E}^{n_{0}}}\delta r_{p_{0m}E}^{n_{0}}+\frac{\partial r_{p_{s}y}^{c}}{\partial \sin\theta_{a}}\delta \sin\theta_{a}+\frac{\partial r_{p_{s}y}^{c}}{\partial \cos\theta_{a}}\delta \cos\theta_{a}$$

(17) $$\begin{array}{ll} \delta r_{p_{s}z}^{c} & =\frac{\partial r_{p_{s}z}^{c}}{\partial r_{p_{s}N}^{n_{0}}}\delta r_{p_{s}N}^{n_{0}}+\frac{\partial r_{p_{s}z}^{c}}{\partial r_{p_{0m}N}^{n_{0}}}\delta r_{p_{0m}N}^{n_{0}}+\frac{\partial r_{p_{s}z}^{c}}{\partial r_{p_{s}E}^{n_{0}}}\delta r_{p_{s}E}^{n_{0}}+\frac{\partial r_{p_{s}z}^{c}}{\partial r_{p_{0m}E}^{n_{0}}}\delta r_{p_{0m}E}^{n_{0}}+\frac{\partial r_{p_{s}z}^{c}}{\partial r_{p_{s}D}^{n_{0}}}\delta r_{p_{s}D}^{n_{0}}+\frac{\partial r_{p_{s}z}^{c}}{\partial r_{p_{0m}D}^{n_{0}}}\delta r_{p_{0m}D}^{n_{0}}\\ & +\frac{\partial r_{p_{s}z}^{c}}{\partial \sin\theta_{a}}\delta \sin\theta_{a}+\frac{\partial r_{p_{s}z}^{c}}{\partial \cos\theta_{a}}\delta \cos\theta_{a}+\frac{\partial r_{p_{s}z}^{c}}{\partial \sin\theta_{l}}\delta \sin\theta_{l}+\frac{\partial r_{p_{s}z}^{c}}{\partial \cos\theta_{l}}\delta \cos\theta_{l} \end{array}$$

**Figure 7:**
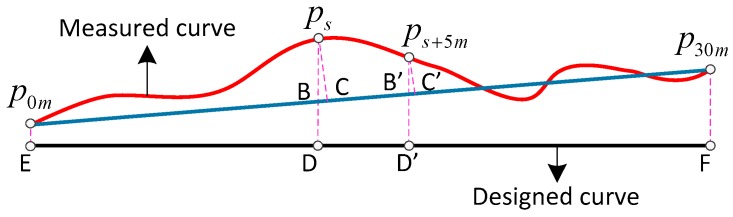
The relationship between absolute deviation and relative deviation.

Substituting Equations (16) and (17) into Equation (1) yields the alignment irregularity and the level irregularity. Since gradient of the railway track is very small (25 m/1000 m for the largest gradient) and the turning radius is very large (2000 m) in general, the alignment irregularity and the level irregularity can be simplified by ignoring the small terms as Equations (18) and (19) show:

(18) $$\begin{array}{ll} \Delta_{p_{s}y} & =\delta r_{p_{s}y}^{c}-\delta r_{p_{s+5m}y}^{c}\\ & \approx -\left[\delta r_{p_{s}N}^{n_{0}}-\delta r_{p_{s+5m}N}^{n_{0}}+\frac{l_{5m}}{l_{c}}\left(\delta r_{p_{30m}N}^{n_{0}}-\delta r_{p_{0m}N}^{n_{0}}\right)\right]\sin\theta_{a}+\left[\delta r_{p_{s}E}^{n_{0}}-\delta r_{p_{s+5m}E}^{n_{0}}+\frac{l_{5m}}{l_{c}}\left(\delta r_{p_{30m}E}^{n_{0}}-\delta r_{p_{0m}E}^{n_{0}}\right)\right]\cos\theta_{a} \end{array}$$

(19) $$\Delta_{p_{s}z}=\delta r_{p_{s}z}^{c}-\delta r_{p_{s+5m}z}^{c}\approx \left(\delta r_{p_{s}D}^{n_{0}}-\delta r_{p_{s+5m}D}^{n_{0}}\right)+\frac{l_{5m}}{l_{c}}\left(\delta r_{p_{30m}D}^{n_{0}}-\delta r_{p_{0m}D}^{n_{0}}\right)$$

where $l_{c}=\sqrt{{\left(r_{p_{30m}N}^{n_{0}}-r_{p_{0m}N}^{n_{0}}\right)}^{2}+{\left(r_{p_{30m}E}^{n_{0}}-r_{p_{0m}E}^{n_{0}}\right)}^{2}+{\left(r_{p_{30m}D}^{n_{0}}-r_{p_{0m}D}^{n_{0}}\right)}^{2}}$ represents the length of the chord. $l_{5m}=5m$ is the distance between points $p_{s}$ and $p_{s+5m}$. The derivation processes of Equations (18) and (19) are shown in Appendix A. As Figure 7 illustrates, even though the absolute position measurements may have deviations bigger than centimeter scale, the relative deviations can also be millimeter scale due to the common offset contained by the adjacent points $p_{s}$ and $p_{s+5m}$.

## 5. Covariance Analysis

The track irregularity measurement accuracy can be presented by its variance. According to Equations (18) and (19), in order to calculate the variance of track irregularity, the covariances among $\delta r_{p_{i}N}^{n_{0}}$ $\delta r_{p_{i}E}^{n_{0}}$ and $\delta r_{p_{i}D}^{n_{0}}$ (i=0 m,s,s+5 m,30 m) should be calculated. However, the analytical solutions of the Riccati equation and the state vector of the filtering and smoothing system are difficult to calculate, we will firstly carry out the covariance analysis theoretically for the simplified situation by ignoring the system noises, and numerically for the general situation with system noises. Since the projections of the Earth angular velocity through the attitude errors are small and remain constant in short time interval, they can be equivalent to the gyro drifts. The terms of Coriolis acceleration are so small that they can also be ignored in a short time interval. Therefore, the system matrix can be further simplified as expressed in Equation (20) for the simplified situation [10]:

(20) $$A=\left[\begin{array}{cccccccccccc} 0 & 0 & 0 & 0 & 0 & 0 & 0 & 0 & 0 & -1 & 0 & 0\\ 0 & 0 & -\Omega \cos L & 0 & 0 & 0 & 0 & 0 & 0 & 0 & 0 & 0\\ 0 & 0 & 0 & 0 & 0 & 0 & 0 & 0 & 0 & 0 & -1 & 0\\ 0 & -g & 0 & 0 & 0 & 0 & 0 & 0 & 0 & 0 & 0 & 0\\ g & 0 & 0 & 0 & 0 & 0 & 0 & 0 & 0 & 0 & 0 & 0\\ 0 & 0 & 0 & 0 & 0 & 0 & 0 & 0 & 0 & 0 & 0 & 1\\ 0 & 0 & 0 & 1 & 0 & 0 & 0 & 0 & 0 & 0 & 0 & 0\\ 0 & 0 & 0 & 0 & 1 & 0 & 0 & 0 & 0 & 0 & 0 & 0\\ 0 & 0 & 0 & 0 & 0 & 1 & 0 & 0 & 0 & 0 & 0 & 0\\ 0 & 0 & 0 & 0 & 0 & 0 & 0 & 0 & 0 & 0 & 0 & 0\\ 0 & 0 & 0 & 0 & 0 & 0 & 0 & 0 & 0 & 0 & 0 & 0\\ 0 & 0 & 0 & 0 & 0 & 0 & 0 & 0 & 0 & 0 & 0 & 0 \end{array}\right]$$

where g is the value of gravity. In addition, in order to simplify the solving process, we suppose that the railway track is a straight track in north direction without loss of generality. In these conditions, Equations (18) and (19) can be simplified as expressed by Equations (21) and (22):

(21) $$\Delta_{p_{s}y}=\delta r_{p_{s}y}^{c}-\delta r_{p_{s+5m}y}^{c}=\left(\delta r_{p_{s}E}^{n_{0}}-\delta r_{p_{s+5m}E}^{n_{0}}\right)+\frac{l_{5m}}{l_{c}}\left(\delta r_{p_{30m}E}^{n_{0}}-\delta r_{p_{0m}E}^{n_{0}}\right)$$

(22) $$\Delta_{p_{s}z}=\delta r_{p_{s}z}^{c}-\delta r_{p_{s+5m}z}^{c}=\left(\delta r_{p_{s}D}^{n_{0}}-\delta r_{p_{s+5m}D}^{n_{0}}\right)+\frac{l_{5m}}{l_{c}}\left(\delta r_{p_{30m}D}^{n_{0}}-\delta r_{p_{0m}D}^{n_{0}}\right)$$

For a ZUPT-aided INS with landmark integration, the distribution of trolley stop points and position observations is crucial to ensure the surveying accuracy [5]. In general, higher-frequency observation updates will result in better accuracy. However higher-frequency observation means more stop points which will influence the work efficiency. Therefore, in this paper, the distance of two stop points is 60 m for measuring position and providing zero velocity, and the observation updates are only provided in the end of every 30 m chord interval as Figure 6 shows.

For the measurement of every 30 m interval, the observation updates are measured at the end time epoch. For the forward filtering process, the optimal estimate of error state vector and its covariance matrix at other time epochs with no observations can be expressed as the functions of initial values in continuous form as Equation (23) shows according to Equation (11) [13]:

(23) $$\begin{array}{l} {\hat{x}}_{f}\left(t\right)=\Phi \left(t,0\right){\hat{x}}_{f}\left(0\right)\\ P_{f}\left(t\right)=\Phi \left(t,0\right)P_{f}\left(0\right)\Phi^{T}\left(t,0\right) \end{array}$$

where Φ(t,0) is the system state transition matrix can be calculated as Equation (24) shows:

(24) $$\Phi \left(t,0\right)=e^{At}=\left[\begin{array}{cccccccccccc} 1 & 0 & 0 & 0 & 0 & 0 & 0 & 0 & 0 & -t & 0 & 0\\ 0 & 1 & \Omega t\cos L & 0 & 0 & 0 & 0 & 0 & 0 & 0 & -\frac{\Omega t^{2}\cos L}{2} & 0\\ 0 & 0 & 1 & 0 & 0 & 0 & 0 & 0 & 0 & 0 & -t & 0\\ 0 & -gt & -\frac{\Omega t^{2}\cos L}{2} & 1 & 0 & 0 & 0 & 0 & 0 & 0 & \frac{g\Omega t^{3}\cos L}{6} & 0\\ gt & 0 & 0 & 0 & 1 & 0 & 0 & 0 & 0 & -\frac{gt^{2}}{2} & 0 & 0\\ 0 & 0 & 0 & 0 & 0 & 1 & 0 & 0 & 0 & 0 & 0 & t\\ 0 & -\frac{gt^{2}}{2} & -\frac{g\Omega t^{3}\cos L}{6} & t & 0 & 0 & 1 & 0 & 0 & 0 & \frac{g\Omega t^{4}\cos L}{24} & 0\\ \frac{gt^{2}}{2} & 0 & 0 & 0 & t & 0 & 0 & 1 & 0 & -\frac{gt^{2}}{6} & 0 & 0\\ 0 & 0 & 0 & 0 & 0 & t & 0 & 0 & 1 & 0 & 0 & \frac{t^{2}}{2}\\ 0 & 0 & 0 & 0 & 0 & 0 & 0 & 0 & 0 & 1 & 0 & 0\\ 0 & 0 & 0 & 0 & 0 & 0 & 0 & 0 & 0 & 0 & 1 & 0\\ 0 & 0 & 0 & 0 & 0 & 0 & 0 & 0 & 0 & 0 & 0 & 1 \end{array}\right]$$

and the initial variance matrix can be expressed as Equation (25):

(25) $$P\left(0\right)=diag\left(\left[\begin{array}{llllllllllll} p_{\phi_{N}} & p_{\phi_{E}} & p_{\phi_{D}} & p_{\delta v_{N}} & p_{\delta v_{E}} & p_{\delta v_{D}} & p_{\delta r_{N}} & p_{\delta r_{E}} & p_{\delta r_{D}} & p_{\varepsilon_{N}} & p_{\varepsilon_{D}} & p_{\nabla_{D}} \end{array}\right]\right)$$

When the observations update at time epoch T, the updated estimate of state vector and its covariance can be obtained by disperse Kalman filter as shown in Equation (26):

(26) $$\begin{array}{ll} {\hat{x}}_{fN}^{+} & ={\hat{x}}_{fN}^{-}+K_{N}\left({\tilde{z}}_{N}-H_{N}{\hat{x}}_{fN}^{-}\right)\\ & =\Phi \left(T,0\right){\hat{x}}_{f}\left(0\right)+K_{N}H_{N}\Phi \left(T,0\right)\left[x\left(0\right)-{\hat{x}}_{f}\left(0\right)\right]+K_{N}\upsilon_{N}\\ P_{fN}^{+} & =\left(I-K_{N}H_{N}\right)P_{fN}^{-}\\ & =\left(I-K_{N}H_{N}\right)\Phi \left(T,0\right)P_{f}\left(0\right)\Phi^{T}\left(T,0\right) \end{array}$$

For the backward smoothing process, the initial optimal smoothed estimate of state vector and its covariance are $\hat{x}\left(T\right)={\hat{x}}_{fN}^{+}$ and $P\left(T\right)=P_{fN}^{+}$. The optimal smoothed estimate and its covariance at arbitrarily time epoch t can be expressed in continuous form as Equation (27) shows:

(27) $$\begin{array}{l} \hat{x}\left(t\right)=\Phi \left(t,T\right)\hat{x}\left(T\right)\\ P\left(t\right)=\Phi \left(t,T\right)P\left(T\right)\Phi^{T}\left(t,T\right) \end{array}$$

The error of the optimal smoothed estimate of state vector can be obtained by subtracting true value from its optimal smoothed estimate as Equation (28) shows:

(28) $$\begin{array}{ll} \delta x\left(t\right) & =\hat{x}\left(t\right)-x\left(t\right)\\ & =\Phi \left(t,T\right)\hat{x}\left(T\right)-\Phi \left(t,0\right)x\left(0\right)\\ & =\Phi \left(t,T\right)\left[\Phi \left(T,0\right){\hat{x}}_{f}\left(0\right)+K_{N}H_{N}\Phi \left(T,0\right)\left[x\left(0\right)-{\hat{x}}_{f}\left(0\right)\right]+K_{N}\upsilon_{N}\right]-\Phi \left(t,0\right)x\left(0\right)\\ & =\Phi \left(t,0\right)\delta x_{f}\left(0\right)-\Phi \left(t,T\right)K_{N}H_{N}\Phi \left(T,0\right)\delta x_{f}\left(0\right)+\Phi \left(t,T\right)K_{N}\upsilon_{N} \end{array}$$

From Equation (28), we can get the position error $\delta r_{p_{i}N}^{n_{0}}$ $\delta r_{p_{i}E}^{n_{0}}$ and $\delta r_{p_{i}D}^{n_{0}}$ expressed by the initial errors of state vector by setting the corresponding time. Setting $p_{\phi_{N}}=p_{\phi_{E}}=p_{\phi }$, $p_{\delta v_{N}}=p_{\delta v_{E}}=p_{\delta v_{D}}=p_{\delta v}$, $p_{\delta r_{N}}=p_{\delta r_{E}}=p_{\delta r_{D}}=p_{\delta r}$, $p_{\varepsilon_{N}}=p_{\varepsilon_{D}}=p_{\varepsilon }$, $\sigma_{v_{N}}^{2}=\sigma_{v_{E}}^{2}=\sigma_{v_{D}}^{2}=\sigma_{v}^{2}$, $\sigma_{r_{N}}^{2}=\sigma_{r_{E}}^{2}=\sigma_{r_{D}}^{2}=\sigma_{r}^{2}$, $p_{\delta v}=\sigma_{v}^{2}$, and $p_{\delta r}=\sigma_{r}^{2}$. The time of points $p_{s}$ and $p_{s+5m}$ are represented by $t_{s}$ and $t_{s+5m}$, and their relationship is $t_{s+5m}=t_{s}+\frac{l_{5m}}{l_{c}}T=t_{s}+kT$. Substituting $\delta r_{p_{i}E}^{n_{0}}$ and $\delta r_{p_{i}D}^{n_{0}}$ into Equations (21) and (22) respectively and calculating the variances of $\Delta_{p_{s}y}$ and $\Delta_{p_{s}z}$, we can obtain:

(29) $$\begin{array}{ll} P_{\Delta_{p_{s}y}} & =E\left\{\Delta_{p_{s}y}\Delta_{p_{s}y}^{T}\right\}\\ & =\frac{k^{2}g^{2}T^{2}p_{\phi }\sigma_{v}^{2}\left(\sigma_{r}^{2}+9k^{2}\sigma_{v}^{2}T^{2}\right){\left(2t_{s}-5kT\right)}^{2}}{4\left[\sigma_{v}^{2}\sigma_{r}^{2}+9k^{2}\left(\sigma_{v}^{4}+2g^{2}p_{\phi }\sigma_{r}^{2}\right)T^{2}+162k^{4}g^{2}\left(p_{\phi }\sigma_{v}^{2}+p_{\varepsilon }\sigma_{r}^{2}\right)T^{4}+1620k^{6}g^{2}p_{\varepsilon }\sigma_{v}^{2}T^{6}+2916k^{8}g^{4}p_{\phi }p_{\varepsilon }T^{8}\right]}\\ & +\frac{k^{2}g^{2}T^{2}p_{\varepsilon }\sigma_{v}^{2}\left(\sigma_{r}^{2}+9k^{2}\sigma_{v}^{2}T^{2}\right){\left(3t_{s}^{2}+3kTt_{s}-35k^{2}T^{2}\right)}^{2}}{36\left[\sigma_{v}^{2}\sigma_{r}^{2}+9k^{2}\left(\sigma_{v}^{4}+2g^{2}p_{\phi }\sigma_{r}^{2}\right)T^{2}+162k^{4}g^{2}\left(p_{\phi }\sigma_{v}^{2}+p_{\varepsilon }\sigma_{r}^{2}\right)T^{4}+1620k^{6}g^{2}p_{\varepsilon }\sigma_{v}^{2}T^{6}+2916k^{8}g^{4}p_{\phi }p_{\varepsilon }T^{8}\right]}\\ & +\frac{k^{4}g^{4}T^{4}p_{\phi }p_{\varepsilon }\sigma_{r}^{2}{\left(3t_{s}^{2}-15kTt_{s}+10k^{2}T^{2}\right)}^{2}}{2\left[\sigma_{v}^{2}\sigma_{r}^{2}+9k^{2}\left(\sigma_{v}^{4}+2g^{2}p_{\phi }\sigma_{r}^{2}\right)T^{2}+162k^{4}g^{2}\left(p_{\phi }\sigma_{v}^{2}+p_{\varepsilon }\sigma_{r}^{2}\right)T^{4}+1620k^{6}g^{2}p_{\varepsilon }\sigma_{v}^{2}T^{6}+2916k^{8}g^{4}p_{\phi }p_{\varepsilon }T^{8}\right]}\\ & +\frac{9k^{6}g^{4}T^{6}p_{\phi }p_{\varepsilon }\sigma_{v}^{2}\left(9t^{4}-90kTt_{s}^{3}+321k^{2}T^{2}t_{s}^{2}-480k^{3}T^{3}t_{s}+325k^{4}T^{4}\right)}{2\left[\sigma_{v}^{2}\sigma_{r}^{2}+9k^{2}\left(\sigma_{v}^{4}+2g^{2}p_{\phi }\sigma_{r}^{2}\right)T^{2}+162k^{4}g^{2}\left(p_{\phi }\sigma_{v}^{2}+p_{\varepsilon }\sigma_{r}^{2}\right)T^{4}+1620k^{6}g^{2}p_{\varepsilon }\sigma_{v}^{2}T^{6}+2916k^{8}g^{4}p_{\phi }p_{\varepsilon }T^{8}\right]} \end{array}$$

(30) $$P_{\Delta_{p_{s}z}}=E\left\{\Delta_{p_{s}z}\Delta_{p_{s}z}^{T}\right\}=\frac{k^{2}p_{\nabla_{D}}\sigma_{v}^{2}T^{2}{\left(5kT-2t_{s}\right)}^{2}}{4\left[\sigma_{v}^{2}+18k^{2}p_{\nabla_{D}}T^{2}\right]}$$

Calculating the partial derivatives about variables of Equations (29) and (30) respectively, we can get $\frac{\partial P_{\Delta_{p_{s}y}}}{\partial p_{\phi }}\geq 0,\frac{\partial P_{\Delta_{p_{s}y}}}{\partial p_{\varepsilon }}\geq 0,\frac{\partial P_{\Delta_{p_{s}y}}}{\partial \sigma_{r}^{2}}\geq 0,\frac{\partial P_{\Delta_{p_{s}y}}}{\partial \sigma_{v}^{2}}>0$ and $\frac{\partial P_{\Delta_{p_{s}z}}}{\partial p_{\nabla_{D}}}\geq 0,\frac{\partial P_{\Delta_{p_{s}z}}}{\partial \sigma_{v}^{2}}\geq 0$. This means that the variances of alignment irregularity and level irregularity are monotone increasing functions. Only with variances about initial state errors and observations less than certain values can the measurements of track irregularity satisfy the surveying accuracy demands. According to Equation (30), the position error ($p_{\delta r}$ and $\sigma_{r}^{2}$) has no effect on level irregularity. As a matter of fact, the influence of position error on the alignment irregularity is also so small than other error terms that can be ignored. When $\sigma_{r}^{2}\to \infty$ which means that there is no position observation, the variance of alignment irregularity can be converted as Equation (31):

(31) $$\begin{array}{ll} \underset{\sigma_{r}^{2}\to \infty }{\lim}P_{\Delta_{p_{s}y}} & =\frac{k^{2}g^{2}T^{2}p_{\phi }\sigma_{v}^{2}{\left(2t_{s}-5kT\right)}^{2}}{4\left(\sigma_{v}^{2}+18k^{2}g^{2}p_{\phi }T^{2}+162k^{4}g^{2}p_{\varepsilon }T^{4}\right)}\\ & +\frac{k^{2}g^{2}T^{2}p_{\varepsilon }\sigma_{v}^{2}{\left(3t_{s}^{2}+3kTt_{s}-35k^{2}T^{2}\right)}^{2}}{36\left(\sigma_{v}^{2}+18k^{2}g^{2}p_{\phi }T^{2}+162k^{4}g^{2}p_{\varepsilon }T^{4}\right)}\\ & +\frac{k^{4}g^{4}T^{4}p_{\phi }p_{\varepsilon }{\left(3t_{s}^{2}-15kTt_{s}+10k^{2}T^{2}\right)}^{2}}{2\left(\sigma_{v}^{2}+18k^{2}g^{2}p_{\phi }T^{2}+162k^{4}g^{2}p_{\varepsilon }T^{4}\right)} \end{array}$$

We can verify that the value of $\underset{\sigma_{r}^{2}\to \infty }{\lim}P_{\Delta_{p_{s}y}}-\underset{\sigma_{r}^{2}\to 0}{\lim}P_{\Delta_{p_{s}y}}$ is very small by a numerical method. Consider that $P_{\Delta_{p_{s}y}}$ is a monotone increasing function of $\sigma_{r}^{2}$, it is feasible to implement track irregularity surveying tasks without position observations updating for the ZUPT-aided INS. As a result, the requirements of high precise landmarks are reduced at a large extent. The landmarks can be only used as a determination of the track segmentation that sub-decimeter scale can meet the demand. And they may be replaced by a sub-decimeter scale INS/odometer integration system in short time interval as well.

For the general situation, the system noises cannot be ignored and we carry out the covariance analysis in a numerical method. The measurement accuracy of alignment irregularity and level irregularity are related with the variances of initial error states, the accuracy of inertial sensors and the accuracy of observation update. Here, we suppose that the railway track is a straight track in the direction of north by east 45 degrees without loss of generality. The system matrix is the full form as Equation (7) shows without ignoring the projections of the Earth angular velocity through the attitude errors and the terms of Coriolis acceleration. Considering the previous analysis, we will only make use of the velocity observation to update the Kalman filter.

**Figure 8:**
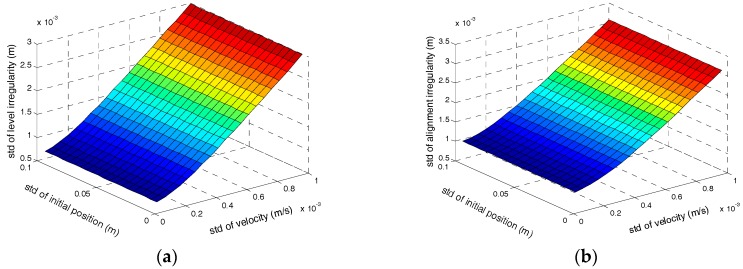
(**a**) The relationship between level irregularity and accuracy of velocity observation as well as initial position; (**b**) The relationship between alignment irregularity accuracy and accuracy of velocity observation as well as initial position.

Firstly, we assess the influences of the observation accuracy on the irregularity measurement accuracy without position observation. Setting the tilt error to 0.006° and the orientation error is 0.06°. Setting the gyro bias instability to 0.01°/h and ARW is 0.005${}^{\circ}/\sqrt{h}$, and setting the accelerometer bias instability to 50 μg and random noise is 10 $\mu g/\sqrt{\mathrm{Hz}}$. The measurement accuracy is also affected by the measurement time of every interval or the velocity of the trolley. Shorter measurement time means less integral time of the errors, and will result in higher measurement accuracy. Here we set the trolley velocity to 1 m/s (8 km/h at most for track surveying trolley), and 30 s will be consumed for every 30 m distance interval. The relationship between railway track irregularities measurement accuracy and the velocity observation accuracy as well as initial position accuracy are illustrated in Figure 8.

According to Figure 8, under the supposed conditions above, the observation accuracy of velocity causes larger influences than the initial position error for the track irregularities measurement accuracy. The initial position error has no effect on the track irregularity, which is coincident with the theoretical analysis previously. In order to satisfy the relative accuracy demand of 1mm, the accuracy of velocity observation should be less than 0.15 mm/s and ZUPT can satisfy the accuracy demand of velocity. A higher level of inertial sensors than the system above should be employed to satisfy the high-speed railway accuracy demand of 0.5 mm.

**Figure 9:**
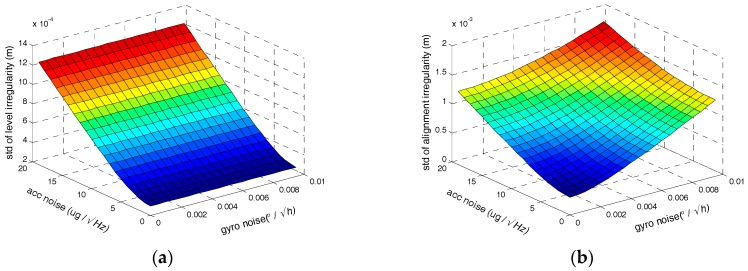
(**a**) The relationship between level irregularity measurement accuracy and noises of gyro and accelerometer; (**b**) The relationship between alignment irregularity measurement accuracy and noises of gyro and accelerometer.

Secondly, we assess the influence of the random noises of inertial sensors on the irregularity measurement accuracy. Setting the tilt error to 0.006° and the orientation error is 0.06°. Setting the gyro bias instability to 0.01°/h, the accelerometer bias instability is 50 μg. and setting the accuracy of initial position to 10 cm the velocity observation is 0.1 mm/s. The relationship between track irregularities measurement accuracy and random noises of gyro and accelerometer are illustrated in Figure 9, where under the supposed conditions above, the ARW of gyro should less than 0.0071${}^{\circ}/\sqrt{h}$ at most and random noise of accelerometer should less than 14.7 $\mu g/\sqrt{\mathrm{Hz}}$ at most to satisfy the demand accuracy of 1 mm both with and without position observation.

**Figure 10:**
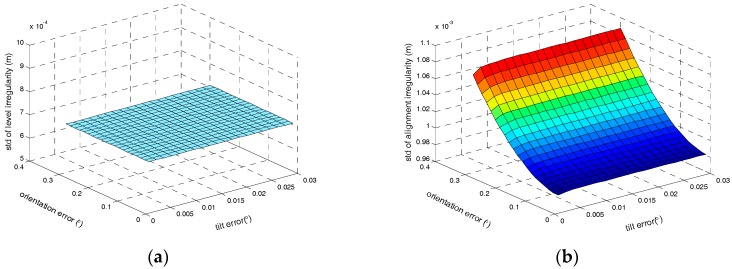
(**a**) The relationship between level irregularity measurement accuracy and attitude errors; (**b**) The relationship between alignment irregularity measurement accuracy and attitude errors.

Thirdly, the influences of the tilt errors and orientation error on the irregularity measurement accuracy have been assessed. Other parameters are fixed as described values previously. The relationship between track irregularities measurement accuracy and attitude errors are illustrated in Figure 10.

As illustrated in Figure 10, the attitude errors have no effect on the level irregularity, which is coincident with the theoretical analysis as Equation (30) shows. Since the orientation error is much bigger, it has a larger effect on alignment irregularity than the tilt errors.

**Figure 11:**
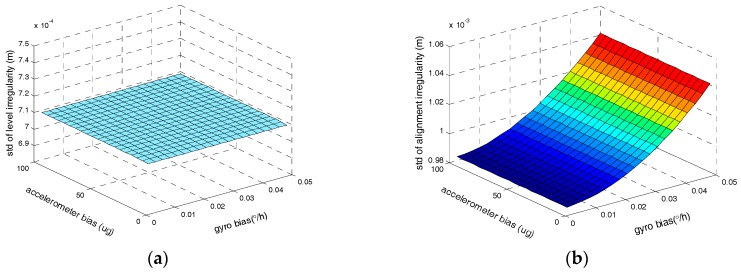
(**a**) The relationship between level irregularity measurement accuracy and the biases of inertial sensors; (**b**) The relationship between alignment irregularity measurement accuracy and the biases of inertial sensors.

Finally, the influences of the equivalent biases of gyros and accelerometers on the irregularities measurement accuracy are assessed as illustrated in Figure 11. Other parameters are also fixed at the previously described values.

As illustrated in Figure 11, the gyro biases have no effect on the level irregularity, which is coincident with the theoretical analysis as shown in Equation (30) and the influence of the accelerometer bias on the level irregularity is small. In addition, the accelerometer bias has no effect on the alignment irregularity as well as Equation (29) shows. 

## 6. Simulations and Experimental Results

### 6.1. Simulations

**Figure 12:**
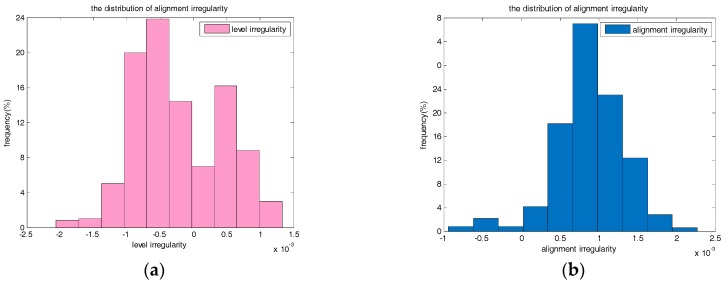
(**a**) The distribution of level irregularity measurement accuracy; (**b**) The distribution of alignment irregularity measurement accuracy.

Monte Carlo simulations of the alignment irregularity and level irregularity surveying accuracy for the proposed approach have been implemented based on the real random noises of INS. The simulated trajectory is a straight line in the direction of north by east 45 degrees. The random noises of gyros and accelerometers are measured by the mentioned INS in static state. The ARW of RLG gyro in this paper is about 0.005${}^{\circ}/\sqrt{h}$, and the bias instability is set to 0.01°/h. The random noise of accelerometer is about 10 $\mu g/\sqrt{\mathrm{Hz}}$, and the bias is set to 50 μg. According to the accuracy of the inertial sensor, the initial attitude errors are set to 0.006° and the initial orientation error is set to 0.06°. The position standard deviations are set to 10 cm, and 0.1 mm/s for the velocity observation. The high precise velocity observation can be provided by ZUPT technique. The velocity of the trolley is set to 1 m/s, and the length of the trajectory is set to 30 m. The observation updates are provided at the beginning and the end of the trajectory. We take the maximum value of track irregularity error to test the statistical accuracy. Five hundred groups of Monte Carlo simulation results based on ZUPT-aided INS approach without position observation are shown in Figure 12.

According to Figure 12, the Root Mean Square (RMS) of measurement accuracy is about 0.70 mm for the level irregularity and 0.99 mm for the alignment irregularity. This is consistent to the result calculated by the covariance analysis previously. The results of Monte Carlo simulation based on ZUPT-aided INS approach with position observation are the same.

### 6.2. Experimental Results

**Figure 13:**
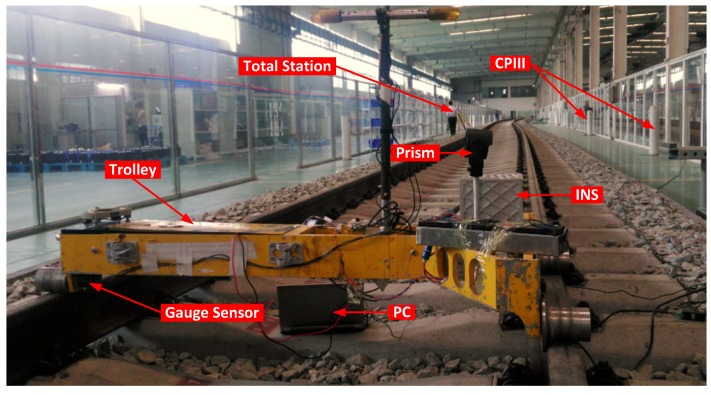
The experimental railway line and the measurement system.

Real tests were carried out on an experimental railway line. The railway track is about 120 m long as shown in Figure 13. The absolute position is provided by a Leica optical total station with a high precision prism mounted on the trolley based on Control Points (CPIII) as shown in the figure.

At the beginning of the tests the trolley is put on the track for 15 min static initial alignment, and loading initial position measured by total station. Then pushing the trolley moves forward on the track at walking speed (about 1.5 m/s) and implementing the measurement of the track irregularities. Two different experiments have been carried out.

**Figure 14:**
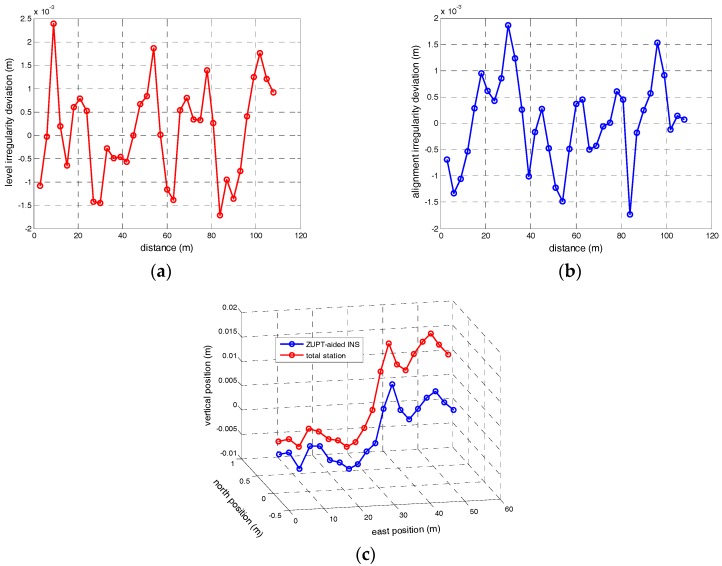
(**a**) Level irregularity deviation for 30 m chord between ZUPT-aided INS and total station; (**b**) Alignment irregularity deviation for 30 m chord between ZUPT-aided INS and total station; (**c**) 3D spatial trajectory of 60 m track segmentation measured by ZUPT-aided INS and total station.

The first experiment is the comparison test of accuracy between the proposed approach and the total station. For this group of tests, the trolley stops at every 60 m distance interval and the velocity observation provided by ZUPT will be updated for the INS. The track irregularities measured by ZUPT-aided INS will compared with the measurements provided by total station. Since the high precise position measurements are measured by total station in every 3 m interval, the distance of two adjacent points calculating the irregularity in Equation (1) is chosen as 6 m. For 30 m chord, the deviation of measurement results between these two approaches is shown in Figure 14. As illustrated, the RMS of alignment irregularity is about 0.82 mm and level irregularity is 1.02 mm. The 3D spatial trajectories of first 60 m track segmentation measured by total station and ZUPT-aided INS are illustrated in Figure 14c. As shown in the figure, even though the absolute deviations between these two approaches are bigger, the relative deviations can still achieve millimeter scale.

**Figure 15:**
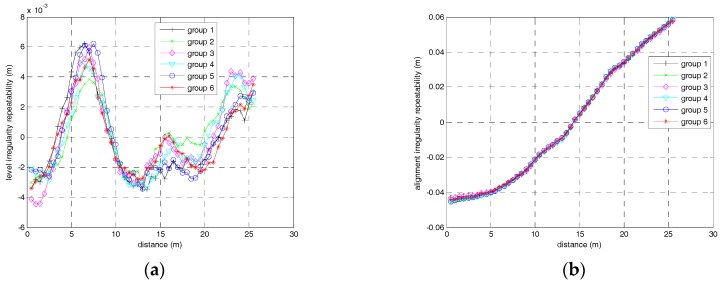
(**a**) Level irregularity measurement repeatability for 30 m chord; (**b**) Alignment irregularity measurement repeatability for 30 m chord.

The second experiment is the repeatability test. For this experiment, six groups of measurements of the same track segment were carried out. Since the designed vector distance is unknown, we just calculate the difference of two points in 5 m intervals, namely ${\tilde{d}}_{s}-{\tilde{d}}_{s+5m}$ to estimate the repeatability of track irregularity. The comparison of track irregularity sequences obtained by ZUPT-aided INS in six runs is illustrated in Figure 15. As shown in the figure, the distance of two adjacent sample points is 0.5 m and only the track irregularities of the first 30 m chord are plotted in the figure.

The irregularity differences at the same railway track points between different runs indicate the repeatability of the measurement. The results of statistic deviation of alignment and level irregularities are listed in Table 1. As Table 1 shows, the standard deviations of differences in alignment irregularity and level irregularity are approximately 1mm, which is consistent with the theoretical analysis as well as the simulation results.

## 7. Conclusions

The measurement of railway track irregularity plays a significant role in monitoring the track deformation and guiding the maintenance of railway lines. This paper makes use of the ZUPT-aided INS for the track irregularity measuring applications. The RTS smoothing algorithm is employed to improve the performance of the surveying system. 

The calculation equations of the track irregularity parameter from absolute positions have been deduced in the paper. Based on covariance analysis, the analytical relationships between the track irregularity with the drifts of inertial sensors, the accuracy of attitude and the accuracy of velocity observations as well as the accuracy of initial position are established. The theoretical analysis and numerical analysis show that the position observation of the Kalman filter has no effect on the measurement accuracy of the alignment irregularity and level irregularity, and we can implement track relative geometry surveying based on ZUPT-aided INS without position observation updates. The landmarks can be only used to determine track segmentation, sub-decimeter scale accuracy of which can satisfy the track surveying demand.

Simulations and experimental results show that the relative accuracy for 30 m chord of the proposed approach for track irregularity surveying can reach approximately 1 mm (1*σ*) with gyro bias instability of 0.01°/h, random walk noise of 0.005${}^{\circ}/\sqrt{h}$ and accelerometer bias instability of 50 μg, random noise of 10 $\mu g/\sqrt{\mathrm{Hz}}$, while only velocity observations are provided by the ZUPT technique in about every 60 m interval. This accuracy can meet the most stringent requirements of the track irregularity surveying for railway lines. For higher accuracy demand of irregularity surveying, the higher level of inertial sensors than that of this paper should be employed.

This paper proposes a relative geometry parameter measuring approach for the railway track. It reduces the requirement of high precision landmarks significantly and lightens the maintenance burden of control points to a large extent. In addition, it also can improve the work efficiency of railway track irregularity measurement task.

## Figures and Tables

**Table 1:** Statistic of alignment irregularity and level irregularity differences.

NO.	Alignment Irregularity (mm)			Level Irregularity (mm)		
	Max	Mean	Std	Max	Mean	Std
G12	3.075	−0.028	1.006	3.854	−0.221	1.296
G13	1.164	0.012	0.445	3.005	−0.131	1.147
G14	1.617	−0.032	0.783	2.954	−0.077	1.082
G15	2.184	−0.088	0.677	2.767	−0.103	0.772
G16	1.403	−0.054	0.527	2.667	−0.071	1.123
G23	3.034	0.040	1.037	2.248	0.089	1.062
G24	1.799	−0.004	0.542	2.250	0.144	0.842
G25	1.340	−0.060	0.654	2.579	0.118	1.161
G26	1.987	−0.026	0.887	2.795	0.150	1.256
G34	1.900	−0.044	0.821	2.192	0.054	1.097
G35	2.143	−0.099	0.707	2.662	0.028	1.104
G36	1.361	−0.066	0.524	3.021	0.060	1.048
G45	1.485	−0.056	0.550	2.947	−0.026	0.914
G46	2.029	−0.022	0.737	3.626	0.006	1.246
G56	1.085	0.034	0.534	2.960	0.032	1.225

Note: G*ij* represents the difference between group *i* and group *j*.

## Appendix A: The derivation processes of Equations (18) and (19).

According to Equation (16), the deviation of $r_{p_{s}y}^{c}$ can be calculated by variational method as expressed by Equation (A1):

(A1) $$\begin{array}{ll} \delta r_{p_{s}y}^{c} & =\frac{\partial r_{p_{s}y}^{c}}{\partial r_{p_{s}N}^{n_{0}}}\delta r_{p_{s}N}^{n_{0}}+\frac{\partial r_{p_{s}y}^{c}}{\partial r_{p_{0m}N}^{n_{0}}}\delta r_{p_{0m}N}^{n_{0}}+\frac{\partial r_{p_{s}y}^{c}}{\partial r_{p_{s}E}^{n_{0}}}\delta r_{p_{s}E}^{n_{0}}+\frac{\partial r_{p_{s}y}^{c}}{\partial r_{p_{0m}E}^{n_{0}}}\delta r_{p_{0m}E}^{n_{0}}+\frac{\partial r_{p_{s}y}^{c}}{\partial \sin\theta_{a}}\delta \sin\theta_{a}+\frac{\partial r_{p_{s}y}^{c}}{\partial \cos\theta_{a}}\delta \cos\theta_{a}\\ & =-\left(\delta r_{p_{s}N}^{n_{0}}-\delta r_{p_{0m}N}^{n_{0}}\right)\sin\theta_{a}+\left(\delta r_{p_{s}E}^{n_{0}}-\delta r_{p_{0m}E}^{n_{0}}\right)\cos\theta_{a}-\left(r_{p_{s}N}^{n_{0}}-r_{p_{0m}N}^{n_{0}}\right)\delta \sin\theta_{a}+\left(r_{p_{s}E}^{n_{0}}-r_{p_{0m}E}^{n_{0}}\right)\delta \cos\theta_{a} \end{array}$$

According to Equation (15), the differential of $\sin\theta_{a}$ and $\cos\theta_{a}$ in Equation (A1) can be derived by Equation (A2):

(A2) $$\begin{array}{l} \delta \sin\theta_{a}=\frac{\partial \sin\theta_{a}}{\partial r_{p_{30m}N}^{n_{0}}}\delta r_{p_{30m}N}^{n_{0}}+\frac{\partial \sin\theta_{a}}{\partial r_{p_{0m}N}^{n_{0}}}\delta r_{p_{0m}N}^{n_{0}}+\frac{\partial \sin\theta_{a}}{\partial r_{p_{30m}E}^{n_{0}}}\delta r_{p_{30m}E}^{n_{0}}+\frac{\partial \sin\theta_{a}}{\partial r_{p_{0m}E}^{n_{0}}}\delta r_{p_{0m}E}^{n_{0}}\\ \delta \cos\theta_{a}=\frac{\partial \cos\theta_{a}}{\partial r_{p_{30m}N}^{n_{0}}}\delta r_{p_{30m}N}^{n_{0}}+\frac{\partial \cos\theta_{a}}{\partial r_{p_{0m}N}^{n_{0}}}\delta r_{p_{0m}N}^{n_{0}}+\frac{\partial \cos\theta_{a}}{\partial r_{p_{30m}E}^{n_{0}}}\delta r_{p_{30m}E}^{n_{0}}+\frac{\partial \cos\theta_{a}}{\partial r_{p_{0m}E}^{n_{0}}}\delta r_{p_{0m}E}^{n_{0}} \end{array}$$

The partial derivative items of Equation (A2) can be expressed as shown by Equations (A3) and (A4):

(A3) $$\begin{array}{l} \frac{\partial \sin\theta_{a}}{\partial r_{p_{30m}N}^{n_{0}}}=-\frac{\left(r_{p_{30m}E}^{n_{0}}-r_{p_{0m}E}^{n_{0}}\right)\left(r_{p_{30m}N}^{n_{0}}-r_{p_{0m}N}^{n_{0}}\right)}{{\left[{\left(r_{p_{30m}N}^{n_{0}}-r_{p_{0m}N}^{n_{0}}\right)}^{2}+{\left(r_{p_{30m}E}^{n_{0}}-r_{p_{0m}E}^{n_{0}}\right)}^{2}\right]}^{\frac{3}{2}}}=-\frac{\sin\theta_{a}\cos\theta_{a}}{\sqrt{{\left(r_{p_{30m}N}^{n_{0}}-r_{p_{0m}N}^{n_{0}}\right)}^{2}+{\left(r_{p_{30m}E}^{n_{0}}-r_{p_{0m}E}^{n_{0}}\right)}^{2}}}\\ \frac{\partial \sin\theta_{a}}{\partial r_{p_{0m}N}^{n_{0}}}=\frac{\sin\theta_{a}\cos\theta_{a}}{\sqrt{{\left(r_{p_{30m}N}^{n_{0}}-r_{p_{0m}N}^{n_{0}}\right)}^{2}+{\left(r_{p_{30m}E}^{n_{0}}-r_{p_{0m}E}^{n_{0}}\right)}^{2}}}\\ \frac{\partial \sin\theta_{a}}{\partial r_{p_{30m}E}^{n_{0}}}=\frac{{\left(r_{p_{30m}N}^{n_{0}}-r_{p_{0m}N}^{n_{0}}\right)}^{2}}{{\left[{\left(r_{p_{30m}N}^{n_{0}}-r_{p_{0m}N}^{n_{0}}\right)}^{2}+{\left(r_{p_{30m}E}^{n_{0}}-r_{p_{0m}E}^{n_{0}}\right)}^{2}\right]}^{\frac{3}{2}}}=\frac{\cos^{2}\theta_{a}}{\sqrt{{\left(r_{p_{30m}N}^{n_{0}}-r_{p_{0m}N}^{n_{0}}\right)}^{2}+{\left(r_{p_{30m}E}^{n_{0}}-r_{p_{0m}E}^{n_{0}}\right)}^{2}}}\\ \frac{\partial \sin\theta_{a}}{\partial r_{p_{0m}E}^{n_{0}}}=-\frac{\cos^{2}\theta_{a}}{\sqrt{{\left(r_{p_{30m}N}^{n_{0}}-r_{p_{0m}N}^{n_{0}}\right)}^{2}+{\left(r_{p_{30m}E}^{n_{0}}-r_{p_{0m}E}^{n_{0}}\right)}^{2}}} \end{array}$$

(A4) $$\begin{array}{l} \frac{\partial \cos\theta_{a}}{\partial r_{p_{30m}N}^{n_{0}}}=\frac{\sin^{2}\theta_{a}}{\sqrt{{\left(r_{p_{30m}N}^{n_{0}}-r_{p_{0m}N}^{n_{0}}\right)}^{2}+{\left(r_{p_{30m}E}^{n_{0}}-r_{p_{0m}E}^{n_{0}}\right)}^{2}}},\;\frac{\partial \cos\theta_{a}}{\partial r_{p_{0m}N}^{n_{0}}}=-\frac{\sin^{2}\theta_{a}}{\sqrt{{\left(r_{p_{30m}N}^{n_{0}}-r_{p_{0m}N}^{n_{0}}\right)}^{2}+{\left(r_{p_{30m}E}^{n_{0}}-r_{p_{0m}E}^{n_{0}}\right)}^{2}}}\\ \frac{\partial \cos\theta_{a}}{\partial r_{p_{30m}E}^{n_{0}}}=-\frac{\sin\theta_{a}\cos\theta_{a}}{\sqrt{{\left(r_{p_{30m}N}^{n_{0}}-r_{p_{0m}N}^{n_{0}}\right)}^{2}+{\left(r_{p_{30m}E}^{n_{0}}-r_{p_{0m}E}^{n_{0}}\right)}^{2}}},\;\frac{\partial \cos\theta_{a}}{\partial r_{p_{30m}E}^{n_{0}}}=\frac{\sin\theta_{a}\cos\theta_{a}}{\sqrt{{\left(r_{p_{30m}N}^{n_{0}}-r_{p_{0m}N}^{n_{0}}\right)}^{2}+{\left(r_{p_{30m}E}^{n_{0}}-r_{p_{0m}E}^{n_{0}}\right)}^{2}}} \end{array}$$

Combining Equations (A2) and (A4) and rearranging yields Equation (A5):

(A5) $$\begin{array}{l} \delta \sin\theta_{a}=-\frac{\left(\delta r_{p_{30m}N}^{n_{0}}-\delta r_{p_{0m}N}^{n_{0}}\right)\sin\theta_{a}\cos\theta_{a}-\left(\delta r_{p_{30m}E}^{n_{0}}-\delta r_{p_{0m}E}^{n_{0}}\right)\cos^{2}\theta_{a}}{\sqrt{{\left(r_{p_{30m}N}^{n_{0}}-r_{p_{0m}N}^{n_{0}}\right)}^{2}+{\left(r_{p_{30m}E}^{n_{0}}-r_{p_{0m}E}^{n_{0}}\right)}^{2}}}\\ \delta \cos\theta_{a}=\frac{\left(\delta r_{p_{30m}N}^{n_{0}}-\delta r_{p_{0m}N}^{n_{0}}\right)\sin^{2}\theta_{a}-\left(\delta r_{p_{30m}E}^{n_{0}}-\delta r_{p_{0m}E}^{n_{0}}\right)\sin\theta_{a}\cos\theta_{a}}{\sqrt{{\left(r_{p_{30m}N}^{n_{0}}-r_{p_{0m}N}^{n_{0}}\right)}^{2}+{\left(r_{p_{30m}E}^{n_{0}}-r_{p_{0m}E}^{n_{0}}\right)}^{2}}} \end{array}$$

Combining Equations (1), (A1) and (A5) and rearranging we can obtain the Equation (A6):

(A6) $$\begin{array}{ll} \Delta_{p_{s}y} & =-\left(\delta r_{p_{s}N}^{n_{0}}-\delta r_{p_{s+5m}N}^{n_{0}}\right)\sin\theta_{a}+\left(\delta r_{p_{s}E}^{n_{0}}-\delta r_{p_{s+5m}E}^{n_{0}}\right)\cos\theta_{a}\\ & +\frac{\left(r_{p_{s}N}^{n_{0}}-r_{p_{s+5m}N}^{n_{0}}\right)\cos\theta_{a}+\left(r_{p_{s}E}^{n_{0}}-r_{p_{s+5m}E}^{n_{0}}\right)\sin\theta_{a}}{\sqrt{{\left(r_{p_{30m}N}^{n_{0}}-r_{p_{0m}N}^{n_{0}}\right)}^{2}+{\left(r_{p_{30m}E}^{n_{0}}-r_{p_{0m}E}^{n_{0}}\right)}^{2}}}\left[\left(\delta r_{p_{30m}N}^{n_{0}}-\delta r_{p_{0m}N}^{n_{0}}\right)\sin\theta_{a}-\left(\delta r_{p_{30m}E}^{n_{0}}-\delta r_{p_{0m}E}^{n_{0}}\right)\cos\theta_{a}\right] \end{array}$$

Considering the small gradient and the large turning radius of the railway track Equation (A6) can be simplified as Equation (18) by ignoring the small terms.

Similarly, the deviation of $r_{p_{s}z}^{c}$ can be calculated by a variational method as expressed by Equation (A7):

(A7) $$\begin{array}{ll} \delta r_{p_{s}z}^{c} & =\frac{\partial r_{p_{s}z}^{c}}{\partial r_{p_{s}N}^{n_{0}}}\delta r_{p_{s}N}^{n_{0}}+\frac{\partial r_{p_{s}z}^{c}}{\partial r_{p_{0m}N}^{n_{0}}}\delta r_{p_{0m}N}^{n_{0}}+\frac{\partial r_{p_{s}z}^{c}}{\partial r_{p_{s}E}^{n_{0}}}\delta r_{p_{s}E}^{n_{0}}+\frac{\partial r_{p_{s}z}^{c}}{\partial r_{p_{0m}E}^{n_{0}}}\delta r_{p_{0m}E}^{n_{0}}+\frac{\partial r_{p_{s}z}^{c}}{\partial r_{p_{s}D}^{n_{0}}}\delta r_{p_{s}D}^{n_{0}}+\frac{\partial r_{p_{s}z}^{c}}{\partial r_{p_{0m}D}^{n_{0}}}\delta r_{p_{0m}D}^{n_{0}}\\ & +\frac{\partial r_{p_{s}z}^{c}}{\partial \sin\theta_{a}}\delta \sin\theta_{a}+\frac{\partial r_{p_{s}z}^{c}}{\partial \cos\theta_{a}}\delta \cos\theta_{a}+\frac{\partial r_{p_{s}z}^{c}}{\partial \sin\theta_{l}}\delta \sin\theta_{l}+\frac{\partial r_{p_{s}z}^{c}}{\partial \cos\theta_{l}}\delta \cos\theta_{l}\\ & =\left(\delta r_{p_{s}N}^{n_{0}}-\delta r_{p_{0m}N}^{n_{0}}\right)\sin\theta_{l}\cos\theta_{a}+\left(\delta r_{p_{s}E}^{n_{0}}-\delta r_{p_{0m}E}^{n_{0}}\right)\sin\theta_{l}\sin\theta_{a}+\left(\delta r_{p_{s}D}^{n_{0}}-\delta r_{p_{0m}D}^{n_{0}}\right)\cos\theta_{l}\\ & +\left(r_{p_{s}N}^{n_{0}}-r_{p_{0m}N}^{n_{0}}\right)\sin\theta_{l}\delta \cos\theta_{a}+\left(r_{p_{s}E}^{n_{0}}-r_{p_{0m}E}^{n_{0}}\right)\sin\theta_{l}\delta \sin\theta_{a}\\ & +\left[\left(r_{p_{s}N}^{n_{0}}-r_{p_{0m}N}^{n_{0}}\right)\cos\theta_{a}+\left(r_{p_{s}E}^{n_{0}}-r_{p_{0m}E}^{n_{0}}\right)\sin\theta_{a}\right]\delta \sin\theta_{l}+\left(r_{p_{s}D}^{n_{0}}-r_{p_{0m}D}^{n_{0}}\right)\delta \cos\theta_{l} \end{array}$$

According to Equation (15), the differential of $\sin\theta_{l}$ and $\cos\theta_{l}$ in Equation (A6) can be derived by Equation (A8):

(A8) $$\begin{array}{ll} \delta \sin\theta_{l}= & \frac{\partial \sin\theta_{l}}{\partial r_{p_{30m}N}^{n_{0}}}\delta r_{p_{30m}N}^{n_{0}}+\frac{\partial \sin\theta_{l}}{\partial r_{p_{0m}N}^{n_{0}}}\delta r_{p_{0m}N}^{n_{0}}+\frac{\partial \sin\theta_{l}}{\partial r_{p_{30m}E}^{n_{0}}}\delta r_{p_{30m}E}^{n_{0}}\\ & +\frac{\partial \sin\theta_{l}}{\partial r_{p_{0m}E}^{n_{0}}}\delta r_{p_{0m}E}^{n_{0}}+\frac{\partial \sin\theta_{l}}{\partial r_{p_{30m}D}^{n_{0}}}\delta r_{p_{30m}D}^{n_{0}}+\frac{\partial \sin\theta_{l}}{\partial r_{p_{0m}D}^{n_{0}}}\delta r_{p_{0m}D}^{n_{0}}\\ \delta \cos\theta_{l}= & \frac{\partial \cos\theta_{l}}{\partial r_{p_{30m}N}^{n_{0}}}\delta r_{p_{30m}N}^{n_{0}}+\frac{\partial \cos\theta_{l}}{\partial r_{p_{0m}N}^{n_{0}}}\delta r_{p_{0m}N}^{n_{0}}+\frac{\partial \cos\theta_{l}}{\partial r_{p_{30m}E}^{n_{0}}}\delta r_{p_{30m}E}^{n_{0}}\\ & +\frac{\partial \cos\theta_{l}}{\partial r_{p_{0m}E}^{n_{0}}}\delta r_{p_{0m}E}^{n_{0}}+\frac{\partial \cos\theta_{l}}{\partial r_{p_{30m}D}^{n_{0}}}\delta r_{p_{30m}D}^{n_{0}}+\frac{\partial \cos\theta_{l}}{\partial r_{p_{0m}D}^{n_{0}}}\delta r_{p_{0m}D}^{n_{0}} \end{array}$$

The partial derivative items of Equation (A8) can be expressed as Equations (A9) and (A10) show:

(A9) $$\begin{array}{l} \frac{\partial \sin\theta_{l}}{\partial r_{p_{30m}N}^{n_{0}}}=\frac{\left(r_{p_{30m}N}^{n_{0}}-r_{p_{0m}N}^{n_{0}}\right)\left(r_{p_{30m}D}^{n_{0}}-r_{p_{0m}D}^{n_{0}}\right)}{{\left[{\left(r_{p_{30m}N}^{n_{0}}-r_{p_{0m}N}^{n_{0}}\right)}^{2}+{\left(r_{p_{30m}E}^{n_{0}}-r_{p_{0m}E}^{n_{0}}\right)}^{2}+{\left(r_{p_{30m}D}^{n_{0}}-r_{p_{0m}D}^{n_{0}}\right)}^{2}\right]}^{\frac{3}{2}}}=-\frac{\left(r_{p_{30m}N}^{n_{0}}-r_{p_{0m}N}^{n_{0}}\right)\sin\theta_{l}}{l_{c}^{2}}\\ \frac{\partial \sin\theta_{l}}{\partial r_{p_{0m}N}^{n_{0}}}=-\frac{\left(r_{p_{30m}N}^{n_{0}}-r_{p_{0m}N}^{n_{0}}\right)\left(r_{p_{30m}D}^{n_{0}}-r_{p_{0m}D}^{n_{0}}\right)}{{\left[{\left(r_{p_{30m}N}^{n_{0}}-r_{p_{0m}N}^{n_{0}}\right)}^{2}+{\left(r_{p_{30m}E}^{n_{0}}-r_{p_{0m}E}^{n_{0}}\right)}^{2}+{\left(r_{p_{30m}D}^{n_{0}}-r_{p_{0m}D}^{n_{0}}\right)}^{2}\right]}^{\frac{3}{2}}}=\frac{\left(r_{p_{30m}N}^{n_{0}}-r_{p_{0m}N}^{n_{0}}\right)\sin\theta_{l}}{l_{c}^{2}}\\ \frac{\partial \sin\theta_{l}}{\partial r_{p_{30m}E}^{n_{0}}}=\frac{\left(r_{p_{30m}E}^{n_{0}}-r_{p_{0m}E}^{n_{0}}\right)\left(r_{p_{30m}D}^{n_{0}}-r_{p_{0m}D}^{n_{0}}\right)}{{\left[{\left(r_{p_{30m}N}^{n_{0}}-r_{p_{0m}N}^{n_{0}}\right)}^{2}+{\left(r_{p_{30m}E}^{n_{0}}-r_{p_{0m}E}^{n_{0}}\right)}^{2}+{\left(r_{p_{30m}D}^{n_{0}}-r_{p_{0m}D}^{n_{0}}\right)}^{2}\right]}^{\frac{3}{2}}}=-\frac{\left(r_{p_{30m}E}^{n_{0}}-r_{p_{0m}E}^{n_{0}}\right)\sin\theta_{l}}{l_{c}^{2}}\\ \frac{\partial \sin\theta_{l}}{\partial r_{p_{0m}E}^{n_{0}}}=-\frac{\left(r_{p_{30m}E}^{n_{0}}-r_{p_{0m}E}^{n_{0}}\right)\left(r_{p_{30m}D}^{n_{0}}-r_{p_{0m}D}^{n_{0}}\right)}{{\left[{\left(r_{p_{30m}N}^{n_{0}}-r_{p_{0m}N}^{n_{0}}\right)}^{2}+{\left(r_{p_{30m}E}^{n_{0}}-r_{p_{0m}E}^{n_{0}}\right)}^{2}+{\left(r_{p_{30m}D}^{n_{0}}-r_{p_{0m}D}^{n_{0}}\right)}^{2}\right]}^{\frac{3}{2}}}=\frac{\left(r_{p_{30m}E}^{n_{0}}-r_{p_{0m}E}^{n_{0}}\right)\sin\theta_{l}}{l_{c}^{2}}\\ \frac{\partial \sin\theta_{l}}{\partial r_{p_{30m}D}^{n_{0}}}=-\frac{{\left(r_{p_{30m}N}^{n_{0}}-r_{p_{0m}N}^{n_{0}}\right)}^{2}+{\left(r_{p_{30m}E}^{n_{0}}-r_{p_{0m}E}^{n_{0}}\right)}^{2}}{{\left[{\left(r_{p_{30m}N}^{n_{0}}-r_{p_{0m}N}^{n_{0}}\right)}^{2}+{\left(r_{p_{30m}E}^{n_{0}}-r_{p_{0m}E}^{n_{0}}\right)}^{2}+{\left(r_{p_{30m}D}^{n_{0}}-r_{p_{0m}D}^{n_{0}}\right)}^{2}\right]}^{\frac{3}{2}}}=-\frac{\cos^{2}\theta_{l}}{l_{c}}\\ \frac{\partial \sin\theta_{l}}{\partial r_{p_{0m}D}^{n_{0}}}=\frac{{\left(r_{p_{30m}N}^{n_{0}}-r_{p_{0m}N}^{n_{0}}\right)}^{2}+{\left(r_{p_{30m}E}^{n_{0}}-r_{p_{0m}E}^{n_{0}}\right)}^{2}}{{\left[{\left(r_{p_{30m}N}^{n_{0}}-r_{p_{0m}N}^{n_{0}}\right)}^{2}+{\left(r_{p_{30m}E}^{n_{0}}-r_{p_{0m}E}^{n_{0}}\right)}^{2}+{\left(r_{p_{30m}D}^{n_{0}}-r_{p_{0m}D}^{n_{0}}\right)}^{2}\right]}^{\frac{3}{2}}}=\frac{\cos^{2}\theta_{l}}{l_{c}} \end{array}$$

(A10) $$\begin{array}{l} \frac{\partial \cos\theta_{l}}{\partial r_{p_{30m}N}^{n_{0}}}=\frac{\cos\theta_{a}\sin^{2}\theta_{l}}{\sqrt{{\left(r_{p_{30m}N}^{n_{0}}-r_{p_{0m}N}^{n_{0}}\right)}^{2}+{\left(r_{p_{30m}E}^{n_{0}}-r_{p_{0m}E}^{n_{0}}\right)}^{2}+{\left(r_{p_{30m}D}^{n_{0}}-r_{p_{0m}D}^{n_{0}}\right)}^{2}}}=\frac{\cos\theta_{a}\sin^{2}\theta_{l}}{l_{c}}\\ \frac{\partial \cos\theta_{l}}{\partial r_{p_{0m}N}^{n_{0}}}=-\frac{\cos\theta_{a}\sin^{2}\theta_{l}}{\sqrt{{\left(r_{p_{30m}N}^{n_{0}}-r_{p_{0m}N}^{n_{0}}\right)}^{2}+{\left(r_{p_{30m}E}^{n_{0}}-r_{p_{0m}E}^{n_{0}}\right)}^{2}+{\left(r_{p_{30m}D}^{n_{0}}-r_{p_{0m}D}^{n_{0}}\right)}^{2}}}=-\frac{\cos\theta_{a}\sin^{2}\theta_{l}}{l_{c}}\\ \frac{\partial \cos\theta_{l}}{\partial r_{p_{30m}E}^{n_{0}}}=\frac{\sin\theta_{a}\sin^{2}\theta_{l}}{\sqrt{{\left(r_{p_{30m}N}^{n_{0}}-r_{p_{0m}N}^{n_{0}}\right)}^{2}+{\left(r_{p_{30m}E}^{n_{0}}-r_{p_{0m}E}^{n_{0}}\right)}^{2}+{\left(r_{p_{30m}D}^{n_{0}}-r_{p_{0m}D}^{n_{0}}\right)}^{2}}}=\frac{\sin\theta_{a}\sin^{2}\theta_{l}}{l_{c}}\\ \frac{\partial \cos\theta_{l}}{\partial r_{p_{0m}E}^{n_{0}}}=-\frac{\sin\theta_{a}\sin^{2}\theta_{l}}{\sqrt{{\left(r_{p_{30m}N}^{n_{0}}-r_{p_{0m}N}^{n_{0}}\right)}^{2}+{\left(r_{p_{30m}E}^{n_{0}}-r_{p_{0m}E}^{n_{0}}\right)}^{2}+{\left(r_{p_{30m}D}^{n_{0}}-r_{p_{0m}D}^{n_{0}}\right)}^{2}}}=-\frac{\sin\theta_{a}\sin^{2}\theta_{l}}{l_{c}}\\ \frac{\partial \cos\theta_{l}}{\partial r_{p_{30m}D}^{n_{0}}}=\frac{\sin\theta_{l}\cos\theta_{l}}{\sqrt{{\left(r_{p_{30m}N}^{n_{0}}-r_{p_{0m}N}^{n_{0}}\right)}^{2}+{\left(r_{p_{30m}E}^{n_{0}}-r_{p_{0m}E}^{n_{0}}\right)}^{2}+{\left(r_{p_{30m}D}^{n_{0}}-r_{p_{0m}D}^{n_{0}}\right)}^{2}}}=\frac{\sin\theta_{l}\cos\theta_{l}}{l_{c}}\\ \frac{\partial \cos\theta_{l}}{\partial r_{p_{0m}D}^{n_{0}}}=-\frac{\sin\theta_{l}\cos\theta_{l}}{\sqrt{{\left(r_{p_{30m}N}^{n_{0}}-r_{p_{0m}N}^{n_{0}}\right)}^{2}+{\left(r_{p_{30m}E}^{n_{0}}-r_{p_{0m}E}^{n_{0}}\right)}^{2}+{\left(r_{p_{30m}D}^{n_{0}}-r_{p_{0m}D}^{n_{0}}\right)}^{2}}}=-\frac{\sin\theta_{l}\cos\theta_{l}}{l_{c}} \end{array}$$

Combining Equations (A9) and (A10) and rearranging yields Equation (A11):

(A11) $$\begin{array}{ll} \delta \sin\theta_{l} & =\frac{\partial \sin\theta_{l}}{\partial r_{p_{30m}N}^{n_{0}}}\delta r_{p_{30m}N}^{n_{0}}+\frac{\partial \sin\theta_{l}}{\partial r_{p_{0m}N}^{n_{0}}}\delta r_{p_{0m}N}^{n_{0}}+\frac{\partial \sin\theta_{l}}{\partial r_{p_{30m}E}^{n_{0}}}\delta r_{p_{30m}E}^{n_{0}}\\ & +\frac{\partial \sin\theta_{l}}{\partial r_{p_{0m}E}^{n_{0}}}\delta r_{p_{0m}E}^{n_{0}}+\frac{\partial \sin\theta_{l}}{\partial r_{p_{30m}D}^{n_{0}}}\delta r_{p_{30m}D}^{n_{0}}+\frac{\partial \sin\theta_{l}}{\partial r_{p_{0m}D}^{n_{0}}}\delta r_{p_{0m}D}^{n_{0}}\\ & =-\frac{\left(r_{p_{30m}N}^{n_{0}}-r_{p_{0m}N}^{n_{0}}\right)\sin\theta_{l}}{l_{c}^{2}}\left(\delta r_{p_{30m}N}^{n_{0}}-\delta r_{p_{0m}N}^{n_{0}}\right)\\ & -\frac{\left(r_{p_{30m}E}^{n_{0}}-r_{p_{0m}E}^{n_{0}}\right)\sin\theta_{l}}{l_{c}^{2}}\left(\delta r_{p_{30m}E}^{n_{0}}-\delta r_{p_{0m}E}^{n_{0}}\right)\\ & -\frac{\cos^{2}\theta_{l}}{l_{c}}\left(\delta r_{p_{30m}D}^{n_{0}}-\delta r_{p_{0m}D}^{n_{0}}\right) \end{array}\;\begin{array}{ll} \delta \cos\theta_{l} & =\frac{\partial \cos\theta_{l}}{\partial r_{p_{30m}N}^{n_{0}}}\delta r_{p_{30m}N}^{n_{0}}+\frac{\partial \cos\theta_{l}}{\partial r_{p_{0m}N}^{n_{0}}}\delta r_{p_{0m}N}^{n_{0}}+\frac{\partial \cos\theta_{l}}{\partial r_{p_{30m}E}^{n_{0}}}\delta r_{p_{30m}E}^{n_{0}}\\ & +\frac{\partial \cos\theta_{l}}{\partial r_{p_{0m}E}^{n_{0}}}\delta r_{p_{0m}E}^{n_{0}}+\frac{\partial \cos\theta_{l}}{\partial r_{p_{30m}D}^{n_{0}}}\delta r_{p_{30m}D}^{n_{0}}+\frac{\partial \cos\theta_{l}}{\partial r_{p_{0m}D}^{n_{0}}}\delta r_{p_{0m}D}^{n_{0}}\\ & =\frac{\cos\theta_{a}\sin^{2}\theta_{l}}{l_{c}}\left(\delta r_{p_{30m}N}^{n_{0}}-\delta r_{p_{0m}N}^{n_{0}}\right)\\ & +\frac{\sin\theta_{a}\sin^{2}\theta_{l}}{l_{c}}\left(\delta r_{p_{30m}E}^{n_{0}}-\delta r_{p_{0m}E}^{n_{0}}\right)\\ & +\frac{\sin\theta_{l}\cos\theta_{l}}{l_{c}}\left(\delta r_{p_{30m}D}^{n_{0}}-\delta r_{p_{0m}D}^{n_{0}}\right) \end{array}$$

By combining Equations (1), (A5), (A7) and (A11) and rearranging we can obtain the Equation (A12):

(A12) $$\begin{array}{ll} \Delta_{p_{s}z} & =\delta r_{p_{s}z}^{c}-\delta r_{p_{s+5m}z}^{c}\\ & =\left(\delta r_{p_{s}N}^{n_{0}}-\delta r_{p_{s+5m}N}^{n_{0}}\right)\sin\theta_{l}\cos\theta_{a}+\left(\delta r_{p_{s}E}^{n_{0}}-\delta r_{p_{s+5m}E}^{n_{0}}\right)\sin\theta_{l}\sin\theta_{a}+\left(\delta r_{p_{s}D}^{n_{0}}-\delta r_{p_{s+5m}D}^{n_{0}}\right)\cos\theta_{l}\\ & +\frac{\left(r_{p_{s}N}^{n_{0}}-r_{p_{s+5m}N}^{n_{0}}\right)\sin\theta_{a}-\left(r_{p_{s}E}^{n_{0}}-r_{p_{s+5m}E}^{n_{0}}\right)\cos\theta_{a}}{\sqrt{{\left(r_{p_{30m}N}^{n_{0}}-r_{p_{0m}N}^{n_{0}}\right)}^{2}+{\left(r_{p_{30m}E}^{n_{0}}-r_{p_{0m}E}^{n_{0}}\right)}^{2}}}\sin\theta_{l}\left[\left(\delta r_{p_{30m}N}^{n_{0}}-\delta r_{p_{0m}N}^{n_{0}}\right)\sin\theta_{a}-\left(\delta r_{p_{30m}E}^{n_{0}}-\delta r_{p_{0m}E}^{n_{0}}\right)\cos\theta_{a}\right]\\ & -\frac{\left(r_{p_{s}N}^{n_{0}}-r_{p_{s+5m}N}^{n_{0}}\right)\cos\theta_{a}+\left(r_{p_{s}E}^{n_{0}}-r_{p_{s+5m}E}^{n_{0}}\right)\sin\theta_{a}}{l_{c}}\frac{\left(r_{p_{30m}N}^{n_{0}}-r_{p_{0m}N}^{n_{0}}\right)}{l_{c}}\left(\delta r_{p_{30m}N}^{n_{0}}-\delta r_{p_{0m}N}^{n_{0}}\right)\sin\theta_{l}\\ & -\frac{\left(r_{p_{s}N}^{n_{0}}-r_{p_{s+5m}N}^{n_{0}}\right)\cos\theta_{a}+\left(r_{p_{s}E}^{n_{0}}-r_{p_{s+5m}E}^{n_{0}}\right)\sin\theta_{a}}{l_{c}}\frac{\left(r_{p_{30m}E}^{n_{0}}-r_{p_{0m}E}^{n_{0}}\right)}{l_{c}}\left(\delta r_{p_{30m}E}^{n_{0}}-\delta r_{p_{0m}E}^{n_{0}}\right)\sin\theta_{l}\\ & -\frac{\left(r_{p_{s}N}^{n_{0}}-r_{p_{s+5m}N}^{n_{0}}\right)\cos\theta_{a}+\left(r_{p_{s}E}^{n_{0}}-r_{p_{s+5m}E}^{n_{0}}\right)\sin\theta_{a}}{l_{c}}\left(\delta r_{p_{30m}D}^{n_{0}}-\delta r_{p_{0m}D}^{n_{0}}\right)\cos^{2}\theta_{l}\\ & +\frac{\left(r_{p_{s}D}^{n_{0}}-r_{p_{s+5m}D}^{n_{0}}\right)}{l_{c}}\left(\delta r_{p_{30m}N}^{n_{0}}-\delta r_{p_{0m}N}^{n_{0}}\right)\cos\theta_{a}\sin^{2}\theta_{l}\\ & +\frac{\left(r_{p_{s}D}^{n_{0}}-r_{p_{s+5m}D}^{n_{0}}\right)}{l_{c}}\left(\delta r_{p_{30m}E}^{n_{0}}-\delta r_{p_{0m}E}^{n_{0}}\right)\sin\theta_{a}\sin^{2}\theta_{l}\\ & +\frac{\left(r_{p_{s}D}^{n_{0}}-r_{p_{s+5m}D}^{n_{0}}\right)}{l_{c}}\left(\delta r_{p_{30m}D}^{n_{0}}-\delta r_{p_{0m}D}^{n_{0}}\right)\sin\theta_{l}\cos\theta_{l} \end{array}$$

Similarly, Equation (A12) can be simplified as Equation (19) by ignoring the small terms.
